# Carbon nanotubes exhibit fibrillar pharmacology in primates

**DOI:** 10.1371/journal.pone.0183902

**Published:** 2017-08-28

**Authors:** Simone Alidori, Daniel L. J. Thorek, Bradley J. Beattie, David Ulmert, Bryan Aristega Almeida, Sebastien Monette, David A. Scheinberg, Michael R. McDevitt

**Affiliations:** 1 Department of Radiology, Memorial Sloan Kettering Cancer Center, New York, New York, United States of America; 2 Departments of Radiology and Radiological Sciences, Johns Hopkins School of Medicine, Baltimore, Maryland, United States of America; 3 Department of Medical Physics, Memorial Sloan Kettering Cancer Center, New York, New York, United States of America; 4 Tri-Instituitional Laboratory of Comparative Pathology, Memorial Sloan Kettering Cancer Center, The Rockefeller University, Weill Cornell Medicine, New York, New York, United States of America; 5 Molecular Pharmacology Program, Memorial Sloan Kettering Cancer Center, New York, New York, United States of America; 6 Department of Pharmacology, Weill Cornell Medicine, New York, New York, United States of America; 7 Department of Medicine, Weill Cornell Medicine, New York, New York, United States of America; 8 Department of Radiology, Weill Cornell Medicine, New York, New York, United States of America; Emory University School of Medicine, UNITED STATES

## Abstract

Nanomedicine rests at the nexus of medicine, bioengineering, and biology with great potential for improving health through innovation and development of new drugs and devices. Carbon nanotubes are an example of a fibrillar nanomaterial poised to translate into medical practice. The leading candidate material in this class is ammonium-functionalized carbon nanotubes (fCNT) that exhibits unexpected pharmacological behavior in vivo with important biotechnology applications. Here, we provide a multi-organ evaluation of the distribution, uptake and processing of fCNT in nonhuman primates using quantitative whole body positron emission tomography (PET), compartmental modeling of pharmacokinetic data, serum biomarkers and ex vivo pathology investigation. Kidney and liver are the two major organ systems that accumulate and excrete [^86^Y]fCNT in nonhuman primates and accumulation is cell specific as described by compartmental modeling analyses of the quantitative PET data. A serial two-compartment model explains renal processing of tracer-labeled fCNT; hepatic data fits a parallel two-compartment model. These modeling data also reveal significant elimination of the injected activity (>99.8%) from the primate within 3 days (t_1/2_ = 11.9 hours). These favorable results in nonhuman primates provide important insight to the fate of fCNT in vivo and pave the way to further engineering design considerations for sophisticated nanomedicines to aid late stage development and clinical use in man.

## Introduction

Nanomaterial medicines, which exist at the interface between matter and life, continue to hold great promise to deliver improvements in patient care. However, limited translation to successful drug candidates or clinical evaluation has become a common refrain. Bioengineering advances in medicinal science unequivocally hinge on performance in vivo [1–3], and ultimately, the complete pharmacological profile of nanomedicines is a crucial property in the translational path to human use. Detailed pharmacokinetic and biocompatibility data obtained in appropriate model organisms permits us to predict human pharmacology. Typically, rodents are widely used to investigate novel materials, however, larger models that more closely parallel human physiology are required to predict translational success and move a field forward.

Biomedical nanocarbon research has realized a plethora of data describing different compositions (i.e., non-covalently or covalently functionalized nanocarbon), each with distinctive pharmacological profiles. Ammonium-functionalized carbon nanotubes have been investigated in rodent models with description of rapid renal clearance and biocompatibility by several different groups that strongly suggest that this embodiment of a water-soluble cylindrical graphene offers a number of distinct advantages for drug delivery [4–14]. Moving forward, nonhuman primates (NHP) provide an extremely important model to investigate the pharmacology of this particular embodiment of fibrillar nanocarbon. The shared lineage and biological similarity of these animals to humans in physiology, development, cognition, neuroanatomy, reproduction, and social complexity permits relevant comparison in the evaluation of novel molecular pharmacological interventions.

The pharmacology of ammonium-functionalized carbon nanotubes in healthy nonhuman primates is described herein. Dynamic positron emission tomography-computed tomography (PET/CT) was leveraged to evaluate the pharmacokinetic profile of tracer-labeled ammonium-functionalized carbon nanotubes using compartmental modeling methods. Blood chemistry, hematology, and clotting analyses along with tissue pathology were used to describe the acute and chronic toxicological profile upon exposure to the nanomaterial. Our data in nonhuman primates reveals that this fibrillar nanocarbon derivative is safe, biocompatible and favorably predicts a pharmacology suitable for translation to man.

## Materials and methods

### Ethics statement

The cynomolgus macaques that participated in this pharmacotoxicology study were housed and cared for in a facility accredited by the Association for Assessment and Accreditation of Laboratory Animal Care International and in compliance with the Animal Welfare Act and the Guide for the Care and Use of Laboratory Animals. All experimental procedures were approved by the MSKCC Institutional Animal Care and Use Committee and in strict accordance with the Animal Welfare Act and the Guide for the Care and Use of Laboratory Animals. Both subjects were compatible and paired continuously. In addition they had visual and auditory access to other macaques 24 hours per day. These animals were fed a complete life-cycle diet (LabDiet, St. Louis, MO) twice daily and fresh produce once daily, with free access to water 24 hours per day. Supplemental food was provided when clinically indicated. Animals were housed in standard stainless steel caging with a minimum floor space of 5.8 square feet per macaque weighing 3.5–5.5 kg. Daily environmental enrichment included rotating manipulanda (forage boards, mirrors, puzzle feeders, etc.) and novel foodstuffs. Under protocol directive, blood was collected from each nonhuman primate and samples were analyzed using an IDEXX Procyte DX Hematology Analyzer (Westbrook, MA) and a Beckman Coulter AU680 Serum Chemistry Analyzer (Brea, CA) by the CCMP Laboratory of Comparative Pathology. Clotting parameters including fibrinogen, using heat precipitation, prothrombin time and partial thromboplastin were measured using a Diagnostica Stago Start 4 instrument and evaluated by IDEXX Laboratories (Grafton, MA). Animals were fasted before procedures requiring anesthesia (ketamine hydrochloride (5–10 mg/kg) +/- dexmedetomidine (0.01–0.02 mg/kg), IM); drinking water was offered ad libitum. At study endpoint monkeys were anesthetized with ketamine hydrochloride + dexmedetomidine injection and then administered an IV injectable commercial euthanasia agent (390 mg pentobarbital (Euthasol, Virbac Animal Health, Fort Worth, TX)). Death was confirmed by a sustained loss of heart beat, a loss of corneal reflexes, and pupillary dilation.

### Synthesis and characterization of ammonium functionalized fibrillar nanocarbon

Single-walled carbon nanotubes (HiPCO, Unidym) were functionalized covalently with multiple copies of primary amines and purified to yield the fibrillar ammonium-functionalized nanocarbon (fCNT) [5,6]. Amine loading per gram of nanocarbon was determined using the Sarin assay [5,6]. Physicochemical characterizations (i.e., dynamic light scattering (DLS), ζ-potential, reverse phase chromatography, and Raman spectroscopy) were performed as described previously in order to validate the identity and purity of the nanomaterial [5,6]. Cryo-transmission electron microscopy (cryoTEM) was performed using a FEI Titan Halo 80–300 kV cryoTEM (Hillsboro, OR) to image the fCNT in solution rather than as a dry solid.

### Radiolabeling fCNT with yttrium-86 (^86^Y)

**Fig 1:**
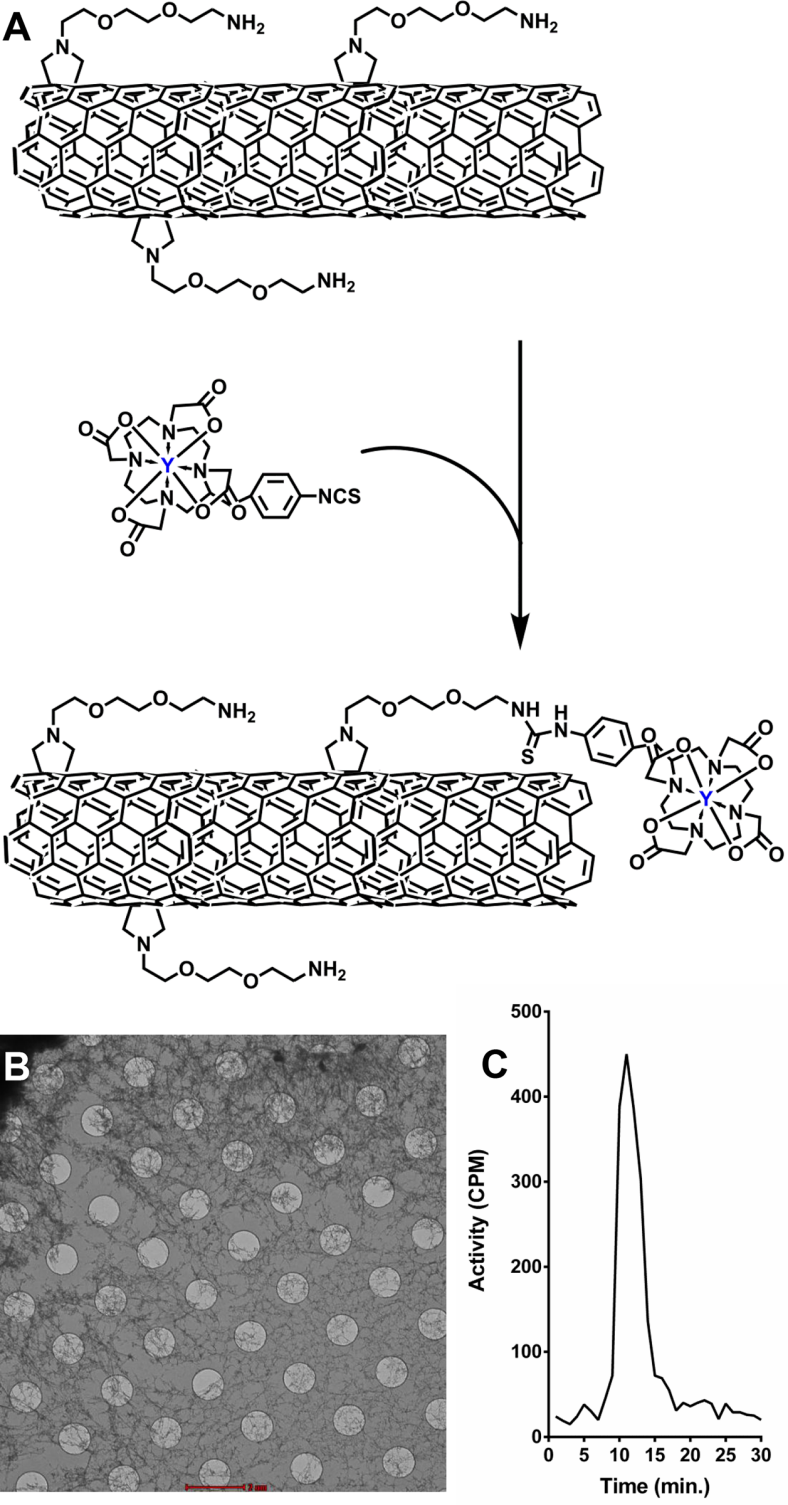
Functionalized fibrillar nanocarbon. (**A**) An illustration of the key moieties covalently appended on the ammonium-functionalized carbon nanotube and the radiosynthetic steps to prepare [^86^Y]fCNT (n.b. not drawn to scale). (**B**) Representative CryoTEM image of fCNT in water (100 μg/L) showing the fibrillar nature of this material in solution (scale bar, 2000 μm). (**C**) Radiochromatograph of [^86^Y]fCNT.

To enable noninvasive real time quantitative analysis of the drug candidate, the fCNT were derivatized with a positron emitting radiolabel. The [^86^Y]fCNT was tracer radiolabeled by reacting 278 MBq (7.5 mCi) of acidic ^86^Y chloride (Washington University) and 2 mg of the radio-metal chelate 2-(4-isothiocyanatobenzyl)-1,4,7,10-tetraazacyclododecane-1,4,7,10-tetraacetic acid (DOTA, Macro-cyclics, Inc.) at pH 5.5 and 60°C for 0.33 hours (Fig 1A). The [^86^Y]DOTA product was then reacted with 12.5 mg of fCNT at pH 9.5 and 37°C for 0.75 hours. The product was purified by size exclusion chromatography using P6 resin (BioRad) as a stationary phase and 1% human serum albumin (HSA, Swiss Red Cross) in 0.9% NaCl (Abbott Laboratories) as mobile phase [5–10]. An aliquot of the [^86^Y]fCNT product was used to determine the radiochemical purity by instant thin layer silica gel (ITLC-SG) chromatography as described [5–10]. Further spectroscopic, radiometric, and chromatographic characterization of the construct was performed by reverse phase HPLC as described [5,6].

### Pharmacokinetic report of [^86^Y]fCNT in mice obtained using tissue harvest

Housing and care for all in vivo experiments were in strict accordance with the Animal Welfare Act and the Guide for the Care and Use of Laboratory Animals; experimental protocols were approved by the MSKCC Institutional Animal Care and Use Committee. Five mice (balb/c, ♀, 2–3 months old, Taconic) each received an intravenous (IV) injection containing 0.03 mg and 74 kBq (0.002 mCi) of [^86^Y]fCNT via the retroorbital sinus. This correlative pharmacokinetic study used the same batch of [^86^Y]fCNT that was used in the primate study. The animals were euthanized 1 hour after administration with CO_2_ aspiration and tissue samples (blood, heart, kidneys, muscle, bone, lung, stomach, liver, spleen, brain and intestine) were harvested, weighed, and counted using a γ-counter (Packard Instrument Co.). Standards of the injected [^86^Y]fCNT were also counted to evaluate the percentage of injected activity per gram of tissue (%IA/g).

### Pharmacokinetic profile of [^86^Y]fCNT in primates acquired from dynamic PET/CT imaging

A Siemens Biograph64 mCT PET/CT was employed to dynamically image two healthy nonhuman primates (*Macaca fascicularis*, ♂, 3.5 years old, Charles River). Each animal received an IV injection containing 5 mg and 5 MBq (0.135 mCi) of [^86^Y]fCNT. All PET images were acquired in time-of-flight mode commencing immediately after injection. Acquisitions began with a 10×1 min. dynamic sequence over the torso (to image heart, liver, kidney and bladder) and then transitioned to a series of whole body scans (n.b., each pass consists of 5 bed positions to image from the head to mid-thigh). The initial 6 passes were acquired at 1 min. per bed; the next 6 at 2 min. per bed; and the final 6 at 3 min. per bed for a total imaging time (including gaps between scans while repositioning the bed) of 195 minutes. Animals were maintained under 2% isoflurane/oxygen anesthesia during the scanning and continually monitored for body temperature, heart rate and respiration. The average decay-corrected activities in the renal, hepatic, and bladder volumes of interest (VOI) were converted to standardized uptake values (SUV) by means of the following calculation: SUV = activity concentration (kBq/mL)/(injection dose (kBq)/body weight (g)). Time-activity curves (TAC) for dynamic analysis were generated using the uptake values for each VOI per frame; SUV values were then plotted as a function of time to describe the kinetics of distribution and clearance. The parameters for quantitative ^86^Y radionuclide PET tracer imaging were previously described [15]. Urine was collected and analyzed by radio-HPLC methods [6].

### Compartmental modeling analysis of [^86^Y]fCNT in primates

The quantitative PET data describing [^86^Y]fCNT uptake into the right kidney cortex and (separately) into liver, were fitted using a linear two-compartment model driven by a blood input forcing function. The model parameters included a blood volume whose value was fixed (15% for kidney, 20% for liver) and four rate constant parameters that were allowed to vary during the fit. For the kidney, the two compartments were assumed to be in a catenary (i.e. serial) configuration, while for the liver a mammillary (i.e. parallel) topology was assumed. Although these topologies are essentially interchangeable, they were selected because could be explained biologically based on data in rodents [6,8]. This interpretation is subject to further investigation. The input function was derived from the dynamic PET images by calculating the mean radioactivity concentration within a VOI placed over the heart. In using this whole-blood input function we have assumed that the entirety of the parent [^86^Y]fCNT in the blood is equally available for uptake into tissue, whether it is bound to plasma proteins or other constituents of the blood, or free in the plasma. The fitting procedure involved a target function that sought to minimize the sum of the weighted squared differences between the model estimate and the corresponding measured values, with weights determined by a model of the noise in each of the PET measures.

### Toxicology of fCNT evaluated from blood chemistry, hematology, and clotting data

Blood was collected from each primate at baseline (4 days prior to injection of [^86^Y]fCNT) and on days 3, 7, 14, 21, 28, 56, 84, 112, 140, 169, and 190 after injection. Blood samples were analyzed using an IDEXX Procyte DX Hematology Analyzer (Westbrook, MA) and a Beckman Coulter AU680 Serum Chemistry Analyzer (Brea, CA) by the MSK Laboratory of Comparative Pathology. Animal 1 was monitored for 6 months after injection to assess acute and chronic changes and Animal 2 was monitored for 2 weeks to investigate acute changes. Renal (serum creatinine (sCr), blood urea nitrogen concentration (BUN), and gamma-glutamyl transpeptidase (GGT)) and hepatic biomarkers (alkaline phosphatase concentration (ALP), alanine aminotransferase concentration (ALT), aspartate aminotransferase concentration (AST), total bilirubin concentration (TBIL), lactate dehydrogenase concentration (LDH), and albumin (ALB)) were evaluated for injury as well as red blood cell count (RBC), white blood cell count (WBC), neutrophils (NEUT), and platelets (PLT). Further tests included a panel of electrolytes (phosphorous (P), calcium (Ca), magnesium (Mg), bicarbonate (TCO2), sodium (Na), and chloride (Cl)), glucose (Glu), triglycerides (TRIG), total cholesterol (CHOL), amylase (AMY), lipase (LIP), total protein (TP), hemoglobin (HGB), hematocrit (HCT), mean corpuscular volume (MCV), mean corpuscular hemoglobin (MCH) and mean corpuscular hemoglobin concentration (MCHC). Clotting parameters including fibrinogen, using heat precipitation, prothrombin time (PT) and partial thromboplastin (PPT) were measured using a Diagnostica Stago Start 4 instrument and evaluated by Idexx Laboratories (Grafton, MA).

### Anatomic pathology following chronic and acute exposure to fCNT

One animal was euthanized for necropsy on day 14 after injection with [^86^Y]fCNT and the other on day 190 to assess acute and chronic effects, respectively. Tissues from a species, age, and gender-matched primate that did not receive [^86^Y]fCNT, retrieved from the archive of the LCP, were evaluated as control. Immediately following euthanasia a complete post-mortem examination, including macroscopic organ examination and dissection was performed. Histologic examination of the following tissues was performed: tracheobronchial lymph node, mesenteric lymph node, submandibular lymph node, lungs, heart, parietal pericardium, aorta, thymus, diaphragm, biceps femoris muscle, parotid salivary gland, submandibular salivary gland, sublingual salivary gland, kidneys, liver and gallbladder, common bile duct, major duodenal papilla, duodenum, spleen, stomach, duodenum, jejunum, ileum, cecum, colon, rectum, pancreas, adrenals, thyroid and parathyroid, trachea, esophagus, urinary bladder, sternum with bone marrow, eye with optic nerve, brain, cervical spinal cord, thoracic spinal cord, lumbar spinal cord, pituitary, sciatic nerve, skin and subcutis, testes and epididymides, prostate, and seminal vesicles. All samples were fixed in 10% neutral buffered formalin, routinely processed in alcohol and xylene, embedded in paraffin, sectioned at 5 μm thickness, and stained with hematoxylin and eosin (H&E). Selected tissues were stained by immunohistochemistry for Iba1 (heat-induced epitope retrieval (HIER) in a citrate based buffer, primary antibody Abcam ab5076 applied at 1:1000 dilution, secondary antibody Vector Labs BA-5000); CD31 (HIER in Dako Target Retrieval Solution S1700, primary Abcam ab187376 at 1:50 dilution, secondary Vector Labs BA-2000); and cleaved caspase-3 ((CC3) HIER pH 6.0, primary Cell Signaling 9661 at 1:250 dilution). Iba1 and CD31 staining was performed manually with an avidin-biotin detection system (Vectastain ABC Elite Kit, Vector Laboratories, PK-6100), and CC3 staining was performed on a Leica Bond RX automated instrument using the Bond Polymer Refine detection system (Leica Biosystem DS9800). These tissues were also stained with the terminal deoxynucleotidyl transferase dUTP nick end labeling (TUNEL) technique, as described previously [8,16]. All slides were evaluated by a board-certified veterinary pathologist (S.M.).

### Lymphatic endothelial cell accumulation of fCNT

Six mice (balb/c, ♀, 2–3 months old, Taconic) were randomly placed into 2 groups (n = 3/group). Mice in Group 1 each received an IV injection containing 0.06 mg of fCNT-AF488 via the retroorbital sinus. Group 2 animals received only the PBS injection vehicle. AlexaFluor 488 tetrafluorophenyl ester (AF488-TFP, Invitrogen), was appended onto fCNT and purified to yield fCNT-AF488 [6,8]. This modification permits us to image fCNT using anti-AF488 immunofluorescence (IF) staining directed at AF488 moieties covalently appended onto the nanocarbon sidewall. One hour after injection the mice were euthanized and lymphatic tissue was harvested, fixed in paraformaldehyde, and embedded in paraffin. Sectioned tissue was stained in the Molecular Cytology Core Facility of MSK using a Discovery XT processor (Ventana Medical Systems). Lymph nodes were stained with 4',6-diamidino-2-phenylindole (DAPI); anti-AF488 antibody (Molecular Probes, cat. no. A-11094, 5 μg/mL); and anti-lymphatic vessel endothelial hyaluronan receptor (Lyve1, R&D Systems, cat. no. AF2125, 1 μg/mL) antibody [8].

### Serum protein binding of fCNT

The dissociation constant between fCNT and human serum albumin (HSA) and human IgG (Bayer, Elkhart, IN) was measured by microscale thermophoresis using a Monolith NT.115 Red/Blue instrument (NanoTemper Technologies, GmbH) at 28°C. The fCNT (1.05 g/L) acts as the fluorescent particle, while the proteins serve as the ligand. HSA was serially diluted from 85.5 g/L to 0.0072 g/L; IgG was serially diluted from 18.5 g/L to 0.0016 g/L. The data were processed and plotted as described [17].

### Water-octanol partition coefficient (K_OW_) of fCNT

The ratio of the concentration of fCNT in an octanol phase was compared to the concentration in an aqueous PBS phase using tracer radiolabeled material. The purity was established using ITLC-SG. Briefly, 2 mL of octanol (Aldrich) and 2 mL of phosphate buffered saline (PBS) were mixed and 0.050 mL of radio-tracer labeled fCNT was added. The sample was mixed by vortex, shaken for 10 minutes, and then centrifuged for 5 minutes at 1000×g to isolate phases. 0.5 mL of each phase was removed and counted using a scintillation counter. A ratio of the cpm per mL in each phase yielded the apparent partition coefficient.

### Data analyses

Three-dimensional region-of-interest analysis on PET images was performed with AsiPRO VM 5.0 (Concorde Microsystems) and in-house software. Model fits to this data were performed using the SAAM II software package (University of Washington, Seattle, WA) and in-house software (developed by B.J.B.). Widefield and confocal microscopy images were evaluated using ImageJ (NIH, http://rsb.info.nih.gov/ij/), AxioVision LE (Zeiss), and Amira 4.1 (Visage Imaging, Inc.) software. Graphs were constructed and statistical data were evaluated using Graphpad Prism 3.0 (Graphpad Software, Inc.). Statistical comparison between 2 experimental groups was performed using a t test (unpaired comparison).

## Results and discussion

### Synthesis and characterization of radiolabeled functionalized carbon nanotubes (fCNT)

The scheme to prepare tracer radiolabeled [^86^Y]fCNT for noninvasive real time quantitative imaging is illustrated in Fig 1A. The chemical synthesis and physicochemical characterization of ammonium-functionalized single-walled fibrillar nanocarbon (fCNT) has been described previously [6,8]. CryoTEM images of fCNT in water (100 μg/L) revealed the fibrillar nature of this material in solution (Fig 1B). fCNT was functionalized with the metal-ion chelate 2-(4-isothiocyanatobenzyl)-1,4,7,10-tetraazacyclododecane-1,4,7,10-tetraacetic acid (DOTA) in order to tracer radiolabel with the positron-emitting yttrium-86 (^86^Y; t_1/2_ = 14.7 h). The [^86^Y]fCNT product was 99.1% radiochemically pure and had a specific activity of 2.55 GBq/g (0.07 Ci/g) at calibration and concentration of 4.9 g/L. Radio-HPLC showed a single peak with a fCNT retention time of 11 minutes (Fig 1C).

**Fig 2:**
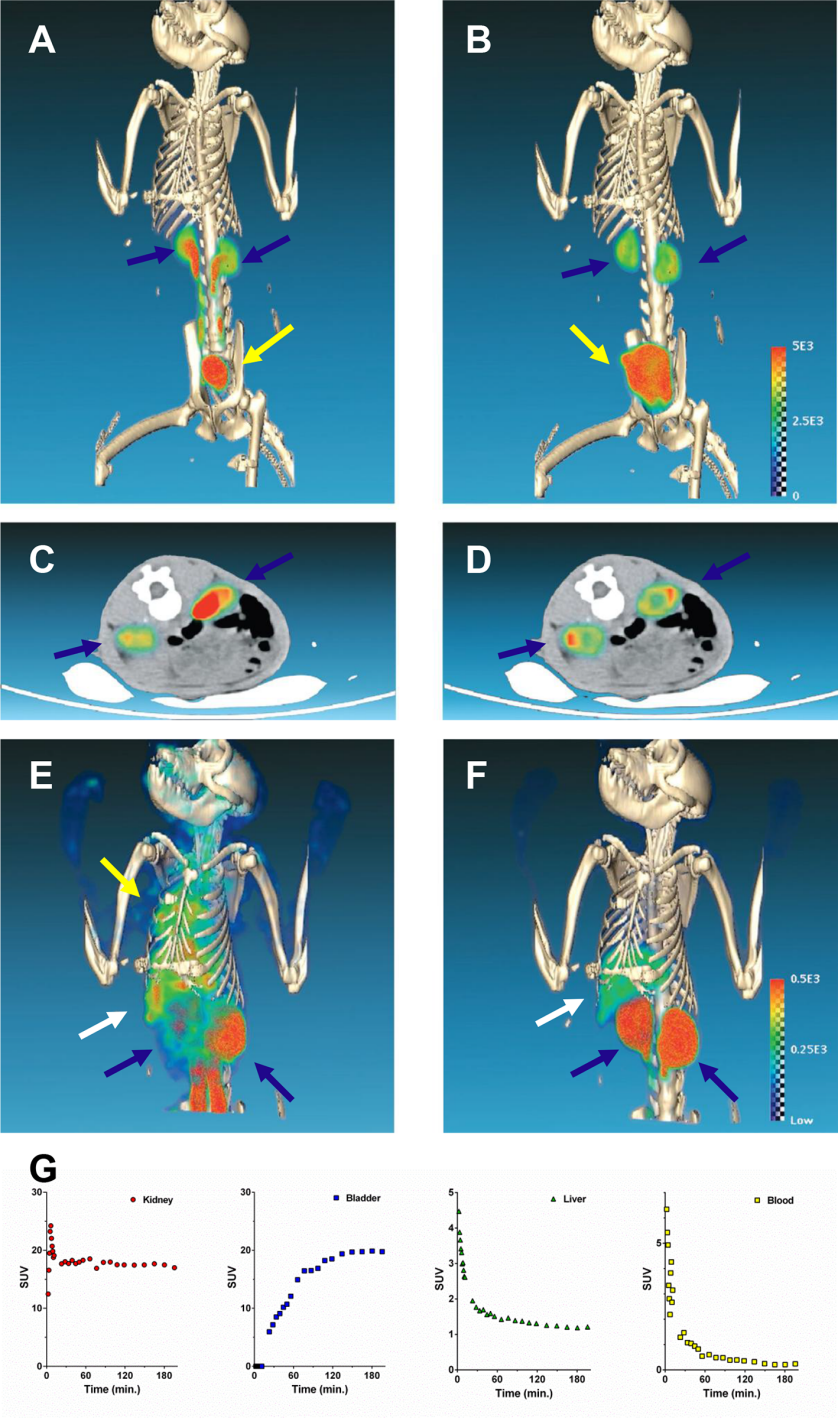
Dynamic PET/CT images of [^86^Y]fCNT in a cynomolgus monkey showing renal and hepatic processing activity. (**A**) Maximum intensity projection (MIP) image immediately following intravenous injection shows rapid kidney accumulation (blue arrows) and elimination of urine into the bladder (yellow arrow); (**B**) MIP of the same animal at 1.5 hours; (**C**) Axial image of the kidneys (blue arrows) immediately following intravenous injection; and (**D**) Axial image of the kidneys at 1.5 hours. (The scale bar is the same for panels **A**-**D**.) (**E**) MIP image immediately following intravenous injection shows diffuse blood compartment and heart activity (yellow arrow) as well as kidney (blue arrow) and liver (white arrow) accumulation of activity; (**F**) MIP of the same animal at 1.5 hours after the blood compartment activity clears and all of the activity has distributed or been eliminated. (The scale bar is the same for panels **E** and **F**.) Time activity curve (TAC) data for (**G**) kidney, urine in bladder, liver, and blood.

fCNT is a covalently functionalized fibrillar nanocarbon. Unlike colloidal detergent-solubilized nanotubes, fCNT is water-soluble (Log P = -3.30) and following intravenous injection rapidly clears the blood compartment undergoing glomerular filtration [5–8] and intact elimination in the urine (Fig 2). This material behaves similarly in rodent and primate models. The renal tissues and proximal tubule cells where this material does accumulate presents unique opportunities for drug delivery as was recently shown with RNA interference directed against the renal injury transcriptome to treat nephropathy [9]. fCNT is a cell-specific drug delivery vehicle that exhibits similar favorable pharmacokinetic and biocompatibility profiles in several model organisms and these new data support translation of fCNT to man.

### The pharmacokinetic profile of [^86^Y]fCNT was measured in rodents

The biodistribution of the tracer [^86^Y]fCNT was confirmed in mice before proceeding to evaluation in nonhuman. Tissue harvest techniques showed that activity was rapidly cleared from the blood within 1 hour (average ± standard deviation was 0.79± 0.35% injected activity per gram (IA/g)) and accumulated predominantly in the kidneys (22.6±5.68%IA/g) and the liver (4.66±0.99%IA/g). The other tissues revealed < 1%IA/g of activity and presumably that contribution was due to the small residual blood compartment activity (S1 Fig). These rodent results are consistent with our previously published data [6,8] and permit us to correlate the performance of the fCNT in the nonhuman primates.

### The pharmacokinetic profile of [^86^Y]fCNT in nonhuman primates

Sedate nonhuman primates were positioned in a clinical hybrid PET/CT scanner, and an initial CT acquisition was acquired. Each subject then received an intravenous injection of [^86^Y]fCNT on camera for a 3 hour dynamic PET protocol. Rapid blood compartment clearance was observed (t_1/2_ ~ 7 min.) and accumulation of activity was imaged in the kidney renal cortices, liver, and the urine collected in the bladder (Fig 2 and S2 Fig). Renal and hepatic time activity curve (TAC) data indicated standard uptake values (SUVs) reached peak values in less that 1 hour for both subjects. In both subjects, the bladder activity increased quickly as any [^86^Y]fCNT that did not accumulate in the cortex was filtered through glomeruli into the urine that collected in the bladder. The urine was collected and analyzed by radio-HPLC to define the pharmacodynamic effects on the construct, if any. This radiochromatograph contained a single peak with a retention time of 11 minutes which matched the retention time of the [^86^Y]fCNT that was injected (S3 Fig) and demonstrated that the labeled fCNT was eliminated intact [5–9].

Quantitative dynamic PET imaging of renal clearance and accretion of activity in the bladder (Fig 2) in both subjects revealed a strong pharmacokinetic correlation with our published mouse data [5–9]. The axial images of fCNT activity distributed in the kidney cortex of these primates has a ring-shaped appearance at 1.5 hours (Fig 2D) and is identical to the pattern observed in mice [5,6]. These axial images describe the elimination of activity from the blood, urine emptied into bladder, and the residual accumulation in cortical tubule cells with a few hours. SUV data shows an activity plateau within 5 minutes in the kidney and by 150 minutes in bladder (Fig 2G and S2 Fig). [^86^Y]fCNT clears intact into urine as was observed in mice [6]. The mouse biodistribution data (S1 Fig) using the same [^86^Y]fCNT test article tested in the NHP produced results in agreement with our previous data [5–9] and correlated well with the primate pharmacokinetic data. This is important as our underlying rodent studies have dissected the unique renal and hepatic processing of fCNT and fully describe the specific cell types (proximal tubule cells and liver sinusoidal endothelium) that accumulate this nanomaterial and explain the mechanisms of renal and hepatic elimination [5–9].

### Renal processing of fCNT in the nonhuman primate

**Fig 3:**
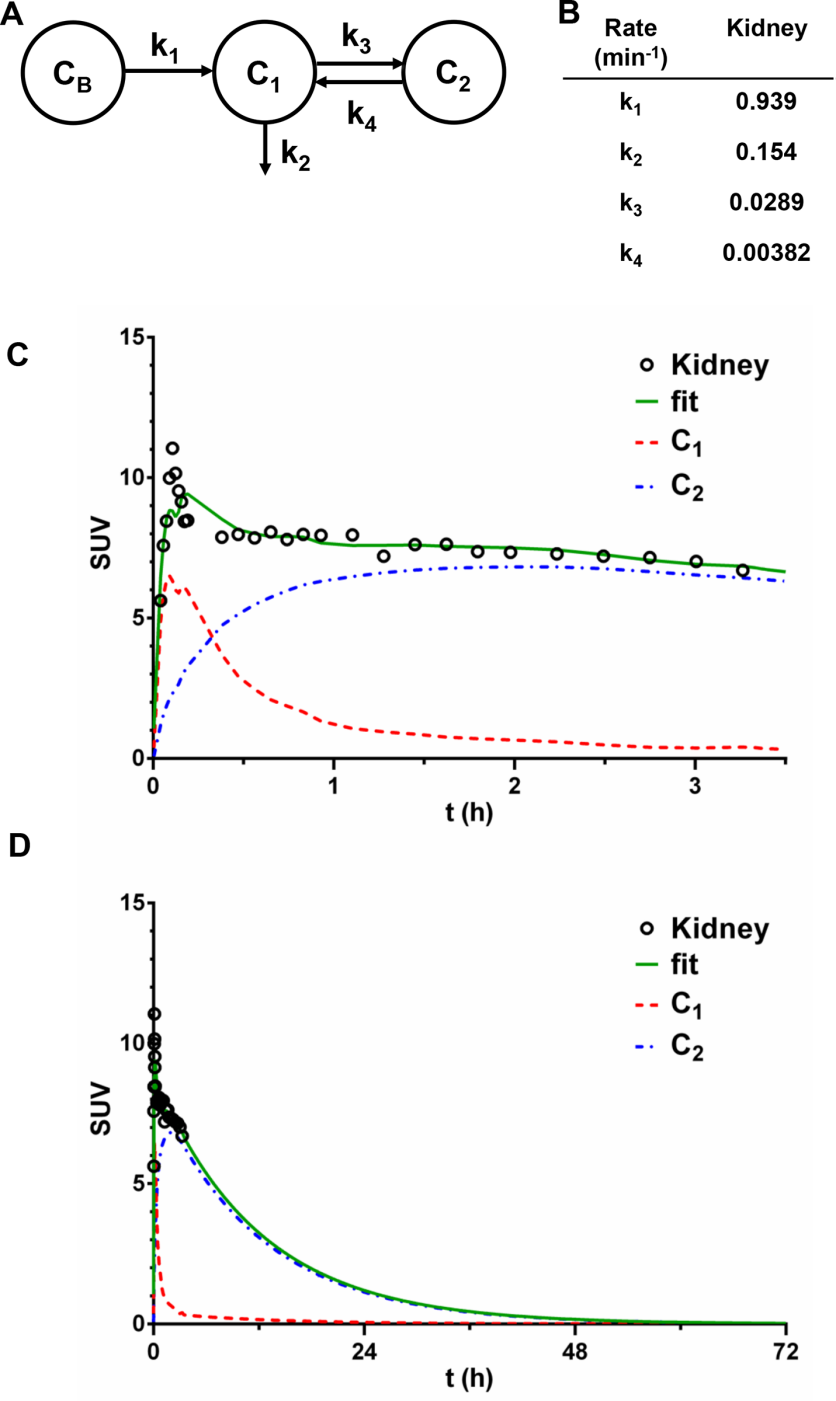
Compartmental modeling analysis of [^86^Y]fCNT pharmacokinetics in the nonhuman primate kidney. The kidney is the primary organ that accumulates and excretes [^86^Y]fCNT in mammals (i.e., primate and rodent) and pharmacokinetically processes [^86^Y]fCNT as described by a serial two-compartment model (**A**). In this model Compartment 1 (C_1_) is assumed to be the renal tissue space *in toto* and Compartment 2 (C_2_) is the proximal tubule cell population that serves as a hold up volume which delays washout from the kidney. This assignment is supported by our data herein (Fig 2) and in rodents [6]. (C_B_) is the blood compartment. The fitted rate constants values are listed in (**B**) where k_1_ denotes transport from blood into kidney; k_3_ indicates the rate constant for accumulation of the tracer in proximal tubule cells; k_4_ describes the fraction of [^86^Y]fCNT that is not accumulated by these target cells, yet remains in kidney and is ultimately eliminated into the urine in a manner described by rate constant k_2_. Plots of the data obtained from quantitative PET/CT imaging and the curve fits are presented in (**C**) and (**D**), where (**D**) draws attention to the significant elimination (99.8%) of the tracer from the kidney within a 72 hour period with a biological half-life of ~12 hours. The standard deviation, coefficient of variance and the 95% confidence intervals for each k value are reported in S1 Table for kidney.

Compartmental modeling analysis of [^86^Y]fCNT pharmacokinetics in the primate yielded key data that reinforces our hypothesis that a fraction of the excreted tracer activity is sequestered in the kidney cortices by renal proximal tubule cells (Figs 2 and 3). Based on our analysis we conjecture that the kidney data fits a serial two-compartment model. Compartment 1 (C_1_) is the renal tissue space *in toto* and Compartment 2 (C_2_) is the proximal tubule cell population that serves as a hold up volume that delays washout from the kidney. Urinary elimination of intact [^86^Y]fCNT is imaged during this PET study. Distribution and clearance of the tracer occurs serially as described by the chart shown in Fig 3A showing the blood compartment (C_B_) and the two tissue compartments (C_1_ and C_2_). The rate constants noted in Fig 3B where k_1_ denotes transport of [^86^Y]fCNT from blood into kidney; k_3_ indicates the rate constant for tracer accumulation in proximal tubule cells of the tracer; k_4_ describes the fraction of tracer that is not accumulated by these target cells, yet remains in kidney and is ultimately eliminated in the urine with a rate constant k_2_. The standard deviation, coefficient of variance and the 95% confidence intervals for each renal k value are reported in S1 Table. Plots of the data obtained from quantitative PET/CT imaging and the curve fits are presented in Fig 3C and 3D for the kidney. The extrapolated fitted curve in Fig 3D draws attention to the significant elimination of 99.8% of tracer from the kidney within a 72 hour period, with a biological half-life of 11.9 hours. This renal cell sink has been described in rodents [5,6,9] and follows from the imaging data (Fig 2) herein. A serial two-compartment model is predicted for the kidneys as fCNT is filtered from the blood (C_B_) at the glomerular interface and a significant fraction is rapidly resorbed by the proximal tubule cells at the luminal brush border; the remaining fraction that is not accumulated exits intact in the urine and collects in the bladder [6]. These data, for the first time, showing that the majority of the activity of fCNT (>99%) is eliminated from the primates in less than 3 days, addresses a long-standing question regarding the persistence of fCNT in mammalian hosts.

### Hepatic processing of fCNT in the nonhuman primate

**Fig 4:**
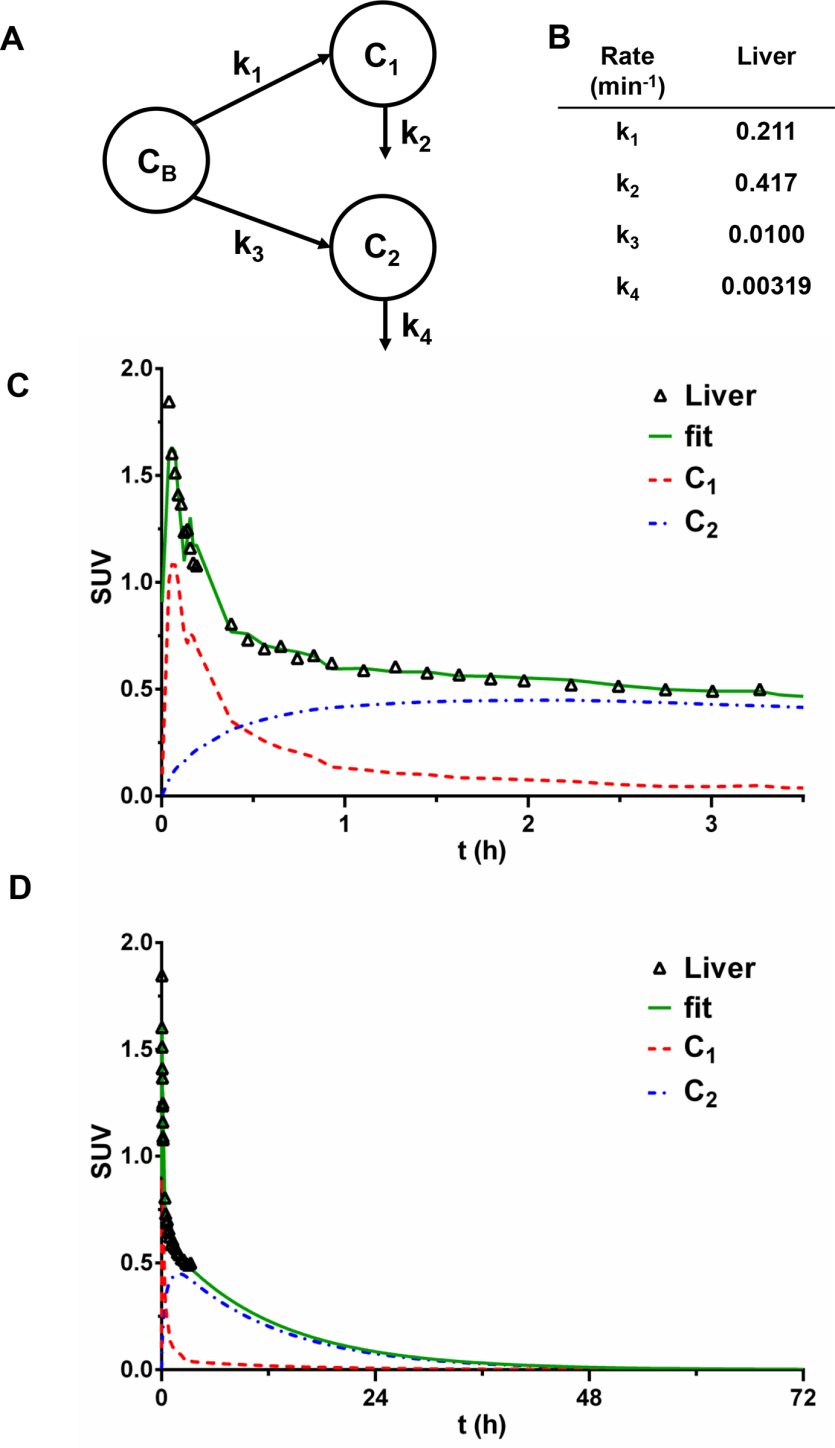
Compartmental modeling analysis of [^86^Y]fCNT pharmacokinetics in the nonhuman primate liver. A parallel two-compartment model is proposed to predict clearance and distribution of [^86^Y]fCNT in the liver, a secondary tissue for processing fCNT. In our model, C_1_ is speculated to be the hepatic tissue space, but excluding the liver sinusoidal endothelium, while C_2_ is assumed to describe specifically the liver sinusoidal endothelium. C_2_ is defined as the compartment having relatively slow clearance, whose correspondence to the sinusoidal endothelium is supported by our data in rodents [8]. Distribution and clearance of the tracer occurs from liver as described by the chart shown in (**A**) showing the blood compartment (C_B_) and the two parallel tissue compartments (C_1_ and C_2_). The rate constants noted in (**B**) where k_1_ denotes transport from blood into C_1_ and k_3_ indicates the rate constant for accumulation of the tracer in liver sinusoidal endothelium. The rate constants k_2_ and k_4_ describe the rate at which [^86^Y]fCNT is returned to the blood and/or eliminated into the bile. Plots of the raw data from PET/CT imaging and the fitted data are presented in (**C**) and (**D**). Note that extrapolation of the fitted data (**D**) calls attention to significant elimination (99.9%) of the tracer from liver within a 72 hour period with biological half-life of ~12 hours. The standard deviation, coefficient of variance and the 95% confidence intervals for each k value are reported in S2 Table for liver.

Liver tissue also realizes a large blood input, yet significantly less activity accumulates compared to the kidney (Figs 2–4). A parallel two-compartment model is proposed to predict clearance and distribution of [^86^Y]fCNT in the liver. In this model, C_1_ is speculated to be the hepatic tissue space *in toto* and C_2_ the liver sinusoidal endothelium serves as another hold up volume. Distribution and clearance of the tracer occurs from liver as described by the chart shown in Fig 4A showing the blood compartment (C_B_) and the two parallel tissue compartments (C_1_ and C_2_). A parallel model is proposed for the liver as the blood borne tracer has equivalent opportunity to partition from blood into C_1_ or C_2_. The rate constants noted in Fig 4B where k_1_ denotes tracer transport from blood into liver and k_3_ indicates the rate constant of accumulation of the tracer in liver sinusoidal endothelium. The rate constants k_2_ and k_4_ describe the fraction of [^86^Y]fCNT that is not accumulated by the liver nor the sinusoidal endothelium and is returned to the blood or eliminated in the bile. The standard deviation, coefficient of variance and the 95% confidence intervals for each k value are reported in S2 Table for liver. Plots of the data from PET/CT imaging and the fitted data are presented in Fig 4C and 4D. Extrapolation of the fitted data (Fig 4D) calls attention to a substantial elimination of the tracer from liver; 99.9% is eliminated within 72 hour from administration with biological half-life of 11.9 hours.

We have reported that the sinusoidal endothelium in liver actively scavenges fCNT from the blood via Stabilin receptors [8] SUV values in the primate liver plateau after 60 minutes as the blood activity clears (Fig 2G). Cynomolgus monkeys express both Stabilin receptors and we presume that the same mechanism of liver accumulation of fCNT is in effect similar to mice [8]. Human liver also expresses this scavenger receptor. A parallel two-compartment model explains the pharmacokinetic fate of the [^86^Y]fCNT tracer in the nonhuman primate liver and is reinforced by our rodent data [8]. We expect that hepatobiliary elimination is a component of the mechanism for elimination from the liver. As with the kidneys, the rate constant describing loss of tracer from C_2_ (k_4_) is an order of magnitude smaller than the entrance rate constant (k_3_) to that compartment indicating concentration of [^86^Y]fCNT in this space.

### Assessing the biocompatibility of fCNT in nonhuman primates using blood markers

**Fig 5:**
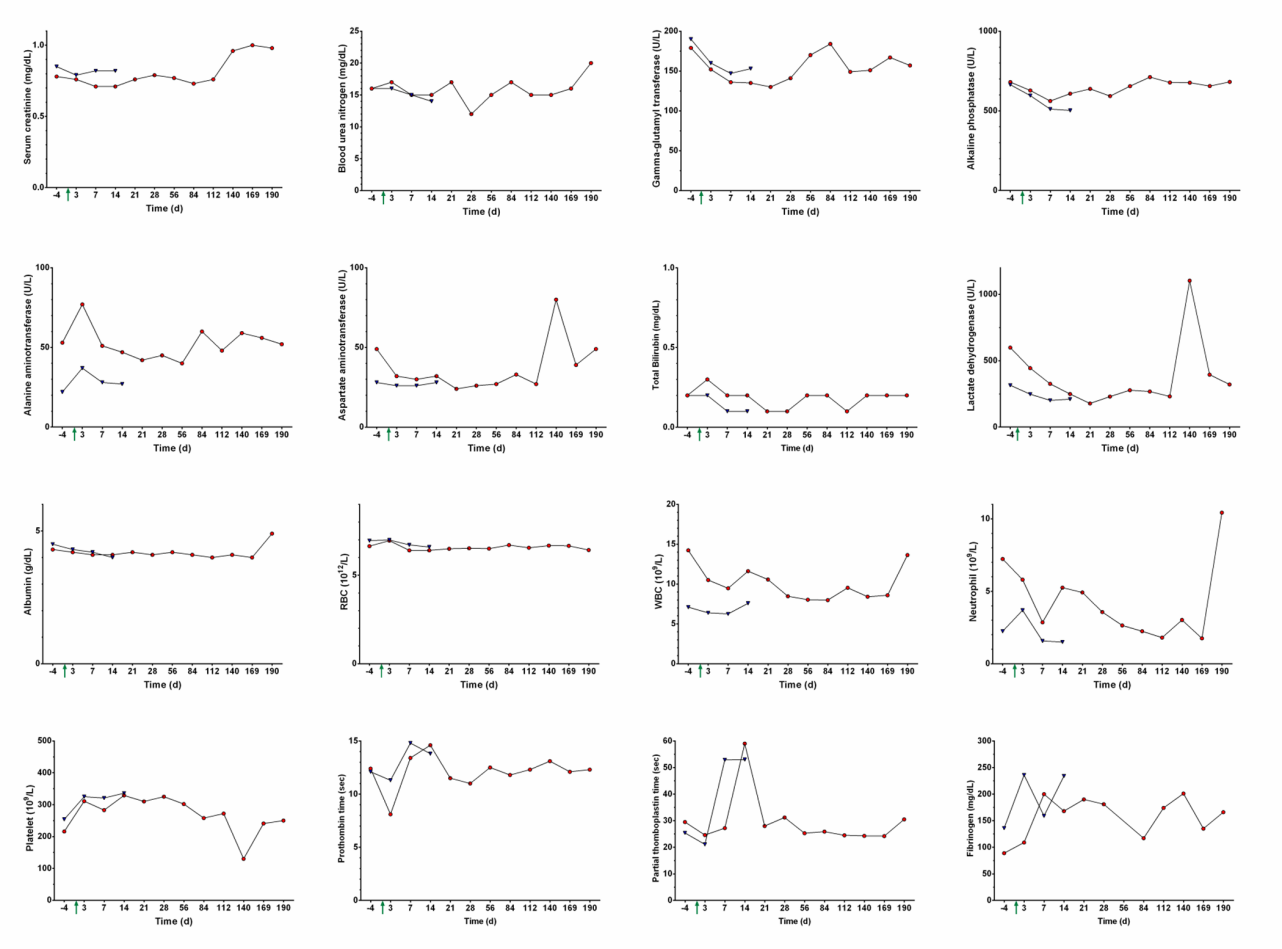
Blood biomarkers, hematology and clotting parameters for primates that received [^86^Y]fCNT. Each cynomolgus monkey received an intravenous dose of [^86^Y]fCNT (1 mg/kg) on day 0 (green arrow indicates time of injection and imaging). Biomarkers for renal and hepatic functions in Animal 1 (red circles) and Animal 2 (blue triangles) were measured at baseline (day minus 4) and longitudinally thereafter until euthanasia and necropsy. Hematology and clotting parameters were also assayed. Additional data can be found in Tables 1 and 2.

Blood samples were analyzed for both animals 4 days prior to injection of [^86^Y]fCNT (baseline) and then longitudinally after infusion until euthanasia and necropsy. Animal 1 was monitored for 6 months after injection to assess acute and chronic changes; Animal 2 was monitored for 2 weeks to measure only acute changes. Renal (serum creatinine (sCr), blood urea nitrogen concentration (BUN), and gamma-glutamyl transpeptidase (GGT)) and hepatic biomarkers (alkaline phosphatase concentration (ALP), alanine aminotransferase concentration (ALT), aspartate aminotransferase concentration (AST), total bilirubin concentration (TBIL), lactate dehydrogenase concentration (LDH), and albumin (ALB)) were evaluated for tissue injury and were stable compared to baseline values and fell within published ranges for similarly aged and gender-matched cynomolgus monkeys [18,19]. Similarly, blood counts (red blood cell count (RBC), white blood cell count (WBC), neutrophils (NEUT), and platelets (PLT)) and RBC volume, shape and hemoglobin characteristics were unchanged from baseline and also within published ranges. Fig 5 and Tables 1 and 2 present the data from these analyses. Interestingly, partial thromboplastin (PPT) values were observed to transiently double after injection, but returned to baseline levels in about two weeks; there were no coagulation issues noted during routine blood analyses. No significant weight loss was noted in either animal following injection (Table 3).

**Table 1:** Blood chemistries and hematology–animal 1.

		Time (d)	-4	3	7	14	21	28	56	84	112	140	169	190
Test	Units													
P	(mg/dL)		5.4	5.6	5.1	4.5	5.3	4.6	5.3	6	5.3	5.8	5.9	4.6
Ca	(mg/dL)		9.5	9.9	9.9	9.6	10.1	9.9	9.8	9.6	9.6	9.4	9.6	9.7
GLU	(mg/dL)		53	68	60	65	69	62	67	69	101	114	107	86
CHOL	(mg/dL)		115	101	85	82	100	97	96	103	99	105	104	112
TRIG	(mg/dL)		47	44	43	67	42	33	72	35	46	51	51	41
CK	(U/L)		333	196	836	206	140	404	211	335	154	8180	689	492
TCO2	(mEq/L)		18	21	23	19	24	20	22	19	25	21	24	22
AMY	(U/L)		150	184	214	183	204	183	146	208	193	157	167	172
LIP	(U/L)		21	8	17	16	10	8	16	20	28	23	30	14
Mg	(mg/dL)		2	1.9	1.9	1.8	1.9	1.9	1.9	1.9	1.8	2	2.1	2.1
Na	(mEq/L)		141	141	142	141	144	143	141	142	138	141	140	145
K	(mEq/L)		4.3	3.5	3.5	3.5	3.5	3.3	3.7	3.5	3.3	4.3	3.8	3.4
Cl	(mEq/L)		104	101	105	106	105	106	103	107	102	105	103	108
Na/K	ratio		33	40	41	40	41	43	38	41	42	33	37	43
Anion Gap			23	23	18	20	19	20	20	20	14	19	17	18
TP	(g/dL)		7.3	7.4	7	6.9	7.2	7.2	7.2	7.2	6.6	6.8	6.9	8.3
ALB	(g/dL)		4.3	4.2	4.1	4.1	4.2	4.1	4.2	4.1	4	4.1	4	4.9
GLOB	(g/dL)		3	3.2	2.9	2.8	3	3.1	3	3.1	2.6	2.7	2.9	3.4
A/G	ratio		1.4	1.3	1.4	1.5	1.4	1.3	1.4	1.3	1.5	1.5	1.4	1.4
RBC	(M/uL)		6.64	6.95	6.4	6.4	6.49	6.52	6.5	6.7	6.55	6.67	6.66	6.42
HGB	(g/dL)		13.4	13.9	12.7	12.8	13.1	13	13.1	13.6	13.2	13.3	13.5	12.9
HCT	(%)		46.8	48.3	44.3	44.9	45.9	46.4	46.2	46	45.7	46	46.4	43.9
MCV	(fL)		70.5	69.5	69.2	70.2	70.7	71.2	71.1	68.7	69.8	69	69.7	68.4
MCH	(pg)		20.2	20	19.8	20	20.2	19.9	20.2	20.3	20.2	19.9	20.3	20.1
MCHC	(g/dL)		28.6	28.8	28.7	28.5	28.5	28	28.4	29.6	28.9	28.9	29.1	29.4
RDW-SD	(fL)		41.4	41	40.3	41	42.6	43	41.6	40.1	41	40	40.8	39.4
RDW-CV	(%)		19.4	19.5	18.5	18.7	19.2	19.4	18.5	19.2	19	18.8	19.1	18.7
RET	(K/uL)		45.2	29.9	23.7	46.1	30.5	36.5	20.8	45.6	33.4	30	34.6	23.8
RET	(%)		0.68	0.43	0.37	0.72	0.47	0.56	0.32	0.68	0.51	0.45	0.52	0.37
PLT	(K/uL)		216	311	283	329	310	325	302	258	272	130	241	250
PDW	(fL)		18.8	13.8	12.7	11.8	12.1	12.6	11.7	12.7	12.3	15.1	12.1	12.1
MPV	(fL)		11.6	11	9.7	9.3	10.1	9.7	9.8	10.5	10.2	11.1	10.4	9.8
P-LCR	(%)		36.9	33.1	24.9	20.8	25.7	24.5	24.5	29.7	27.3	32.9	28	24.7
PCT	(%)		0.25	0.34	0.27	0.3	0.31	0.32	0.3	0.27	0.28	0.14	0.25	0.24
WBC	(K/uL)		14.23	10.5	9.47	11.62	10.57	8.47	8.03	7.98	9.53	8.41	8.59	13.64
NEUT	(K/uL)		7.23	5.8	2.86	5.25	4.92	3.57	2.64	2.24	1.8	3.02	1.75	10.43
LYMPH	(K/uL)		5.8	3.63	5.48	5.2	4.53	3.93	4.51	4.67	6.31	4.55	5.64	2.45
MONO	(K/uL)		1.02	0.92	0.96	1.03	0.94	0.73	0.81	0.94	1.12	0.75	0.97	0.69
EO	(K/uL)		0.17	0.14	0.17	0.14	0.17	0.24	0.07	0.13	0.3	0.09	0.22	0.07
BASO	(K/uL)		0.01	0.01	0	0	0.01	0	0	0	0	0	0.01	0
NEUT	(%)		50.7	55.2	30.2	45.1	46.5	42.2	32.8	28.1	18.9	35.9	20.3	76.4
LYMPH	(%)		40.8	34.6	57.9	44.8	42.9	46.4	56.2	58.5	66.2	54.1	65.7	18
MONO	(%)		7.2	8.8	10.1	8.9	8.9	8.6	10.1	11.8	11.8	8.9	11.3	5.1
EO	(%)		1.2	1.3	1.8	1.2	1.6	2.8	0.9	1.6	3.1	1.1	2.6	0.5
BASO	(%)		0.1	0.1	0	0	0.1	0	0	0	0	0	0.1	0

**Table 2:** Blood chemistries and hematology–animal 2.

		Time (d)	-4	3	7	14
Test	Units					
P	(mg/dL)		5.9	6.2	4.9	5.1
Ca	(mg/dL)		9.6	10.1	10.1	9.7
GLU	(mg/dL)		51	63	62	110
CHOL	(mg/dL)		81	80	71	60
TRIG	(mg/dL)		20	29	19	22
CK	(U/L)		316	203	229	203
TCO2	(mEq/L)		20	21	22	19
AMY	(U/L)		111	142	140	129
LIP	(U/L)		5	3	6	12
Mg	(mg/dL)		1.7	1.7	1.6	1.7
Na	(mEq/L)		143	143	142	141
K	(mEq/L)		3.7	3.6	3.6	3.7
Cl	(mEq/L)		106	105	106	105
Na/K	ratio		39	40	39	38
Anion Gap			21	21	18	21
TP	(g/dL)		7	6.9	6.7	6.3
ALB	(g/dL)		4.5	4.3	4.2	4
GLOB	(g/dL)		2.5	2.6	2.5	2.3
A/G	ratio		1.8	1.7	1.7	1.7
RBC	(M/uL)		6.95	6.99	6.71	6.59
HGB	(g/dL)		12.4	12.5	12	11.7
HCT	(%)		45	44.1	42.8	42.3
MCV	(fL)		64.7	63.1	63.8	64.2
MCH	(pg)		17.8	17.9	17.9	17.8
MCHC	(g/dL)		27.6	28.3	28	27.7
RDW-SD	(fL)		36.2	35.8	35.9	36
RDW-CV	(%)		18.9	18.5	17.8	18.5
RET	(K/uL)		28.5	18.9	21.5	27
RET	(%)		0.41	0.27	0.32	0.41
PLT	(K/uL)		254	325	321	336
PDW	(fL)		18.4	14.9	14.1	16
MPV	(fL)		11.4	10.4	10.2	11.3
P-LCR	(%)		38	30.7	30.1	36.8
PCT	(%)		0.29	0.34	0.33	0.38
WBC	(K/uL)		7.1	6.38	6.25	7.58
NEUT	(K/uL)		2.24	3.69	1.57	1.5
LYMPH	(K/uL)		4.14	2.11	3.82	5.15
MONO	(K/uL)		0.6	0.48	0.7	0.78
EO	(K/uL)		0.12	0.1	0.16	0.15
BASO	(K/uL)		0	0	0	0
NEUT	(%)		31.5	57.8	25.1	19.8
LYMPH	(%)		58.3	33.1	61.1	67.9
MONO	(%)		8.5	7.5	11.2	10.3
EO	(%)		1.7	1.6	2.6	2
BASO	(%)		0	0	0	0

**Table 3:** Body mass of animals 1 and 2.

Date	Animal 1^a^	Animal 2^b^
	(kg)	(kg)
3/26/2014	3.58	3.2
7/8/2014	3.56	3.54
7/22/2014	3.62	3.56
8/5/2014	3.58	3.62
8/19/2014	3.6	3.6
9/2/2014	3.74	3.74
3/23/2015	4.2	4.48
4/13/2015	4.44	4.52
04/17/15^c^	4.41	4.54
4/20/2015	4.22	4.31
4/24/2015	4.41	4.47
5/1/2015	4.3	4.41^d^
5/8/2015	4.38	
5/15/2015	4.42	
6/12/2015	4.54	
7/10/2015	4.8	
10/23/2015	5.20^e^	

^a^Date of birth: 11/18/11

^b^Date of birth: 12/11/11

^c^Date that both animals received [^86^Y]fCNT and were imaged

^d^Date that Animal 2 was euthanized for necropsy

^e^Date that Animal 1 was euthanized for necropsy

Serum biomarkers did not reveal any renal injury (i.e., sCr and BUN) and did not fluctuate outside standard ranges [18,19] following injection of fCNT despite glomerular filtration and tubule cell uptake. Hepatic biomarkers do no indicate any liver injury (i.e., ALP, ALT, AST, TBIL, LDH, and ALB) and fall within the ranges of referenced values [18,19] notwithstanding accumulation of fCNT. These biomarkers are not perturbed by exposure of the liver or kidneys to intravenous fCNT and suggest the absence of injury either acutely or chronically. Similarly, blood cell populations (i.e., RBC, WBC, NEUT, and PLT) were unchanged from baseline and also within published ranges (Fig 5 and Tables 1 and 2). The volume and shape of RBC and both hematocrit and hemoglobin values were stable and do not indicate anemia. Fibrinogen lab values have been reported to vary over a broad range in male cynomolgus monkeys (155–463 mg/dL, n = 50) and our highest values still fall within normal range [18]. The only atypical lab values are PPT that were observed to transiently double after injection, but returned to baseline levels within two weeks. The veterinary staff noted no aberrant coagulation issues during routine blood drawing procedures. PPT times have been reported to vary over a range in male cynomolgus monkeys (15.7–28.0 mg/dL, n = 70) and our highest values doubled in our subjects, but returned to baseline within 2 weeks. Going forward, further testing will focus on the role of diet (i.e., Vitamin K) and repetitive testing to confirm this result. There was no significant weight loss noted in either animal following injection of fCNT (Table 3).

### Anatomic pathology of tissue obtained at necropsy confirmed the biocompatibility of fCNT in nonhuman primates

**Fig 6:**
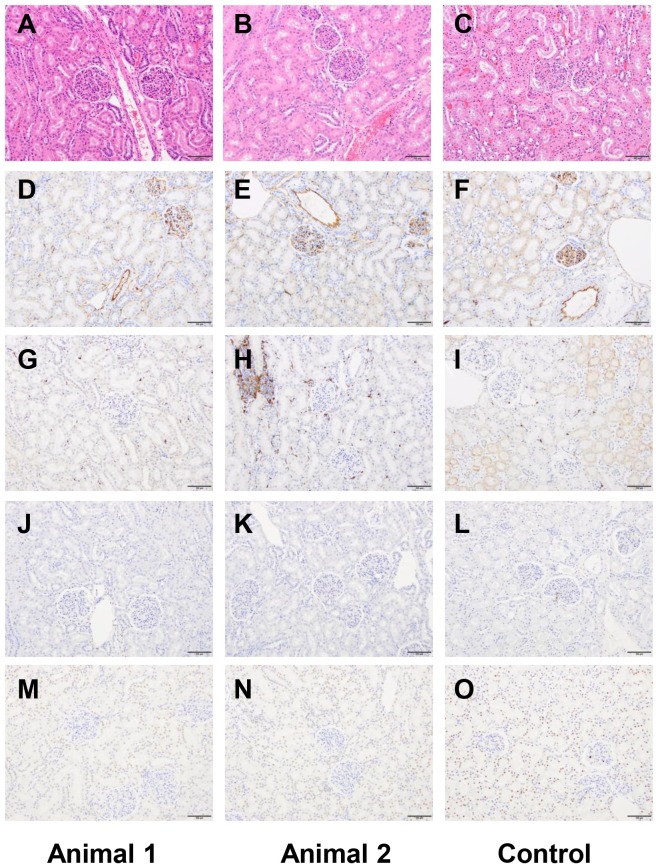
Kidney histology of the two primates that received fCNT and an untreated control. Animal 1 was evaluated at 190 days for chronic effects and Animal 2 was evaluated at 14 days for acute effects (both received [^86^Y]fCNT) and a third animal that did not receive fCNT is an untreated control. Tissue was harvested at necropsy, fixed, sectioned, and stained with (**A-C**) H&E, (**D-F**) anti-CD31, (**G-I**) anti-Iba1, (**J-L**) TUNEL, and (**M-O**) Cleaved caspase 3. All scale bars are 100 μm.

**Fig 7:**
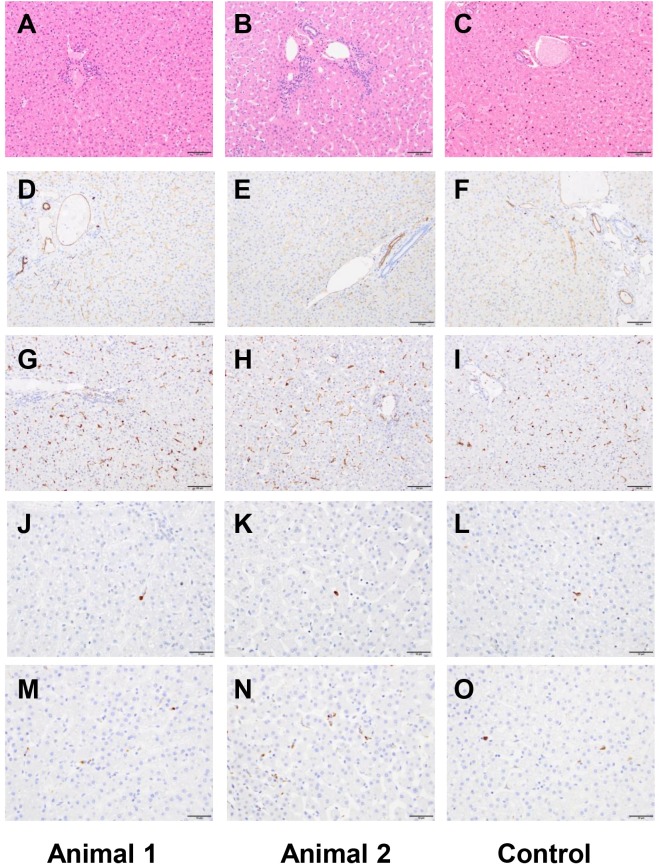
Liver histology of the two primates that received fCNT and an untreated control. Animal 1 was evaluated at 190 days for chronic effects and Animal 2 was evaluated at 14 days for acute effects (both received [^86^Y]fCNT) and a third animal that did not receive fCNT is an untreated control. Tissue was harvested at necropsy, fixed, sectioned, and stained with (**A-C**) H&E, (**D-F**) anti-CD31, (**G-I**) anti-Iba1, (**J-L**) TUNEL, and (**M-O**) Cleaved caspase 3. The scale bars in panels **A-I** are 100 μm; scale bars in panels **J-O** are 50 μm.

A board-certified veterinary pathologist (S.M.) performed both necropsies and reported no remarkable findings in either animal after acute or chronic exposure. A complete panel of tissue was harvested and subsequently analyzed for microscopic effects. We focused additional attention on kidney, liver, spleen and lymph (Figs 6 and 7; S4 and S5 Figs, respectively) examining not only at morphology (H&E) but looked for any evidence of cell death by CC3 and TUNEL and found no differences compared to a historical control primate that did not receive [^86^Y]fCNT. Furthermore, CD31 staining was done to show that the endothelium in these tissues was not perturbed due to treatment. Iba1 staining was also included to evaluate whether macrophage infiltration occurred in tissues that accumulated nanomaterial and this was not evidenced. New data was obtained in mice to confirm the identity of the cell that accumulated fCNT in lymph tissue (S6 Fig). Previously, we had shown conclusively that radiolabeled fCNT was taken up in lymph [8], and this new data imaged tissue that was dual-stained with anti-AF488 (to mark fCNT) and anti-Lyve1 (to mark lymphatic sinusoidal endothelium) to clearly demonstrate co-localization of signals.

Anatomic pathology findings were reported 190 and 14 days post-administration of [^86^Y]fCNT and for Animals 1 and 2, respectively. A board-certified veterinary pathologist (S.M.) concluded that in both subjects there were no significant macroscopic or microscopic abnormalities and the observed changes were almost certainly naturally occurring background lesions. These conclusions are based on i) published pathology data for these animals [20,21], ii) tissue harvested from a control cynomolgus macaques (Figs 6 and 7; S4 and S5 Figs), and iii) practical experience with nonhuman primates in toxicity studies. None of the observed changes appeared to be of clinical significance for the health of the animal.

Representative images of kidney (Fig 6), liver (Fig 7), spleen (S4 Fig) and lymph node (S5 Fig) show tissue stained with H&E, CD31 (endothelial cell marker), Iba1 (macrophage marker), TUNEL (assess cell death) and CC3 (assess apoptosis). These organs were of particular interest because our dynamic PET imaging revealed accumulation of [^86^Y]fCNT in primate kidney and liver (Figs 2–4 and S2 Fig) and also because our previous data in mice has described accumulation of fCNT in the renal cortex and sinusoidal endothelium of liver, spleen, and lymph nodes [6,9]. The uptake of fCNT is cell specific: in the kidney the renal proximal tubule cell population accumulates fCNT via resorption at the brush border from the urine [5,6,9]; in the liver, spleen and lymphatic sinusoids the specialized endothelium that expresses the scavenger Stabilin receptors are the cells that actively endocytose fCNT in rodents [8]. Therefore, we stained for CD31 expression to ask if these endothelial cells were viable and we stained for Iba1 in order to ask if macrophages were recruited in response to fCNT accumulation. In addition, conventional TUNEL and CC3 staining analyses were included to ask if there was cell death. These stains did not reveal any significant changes in treated animals when compared to the tissues of the control animal.

Lymphatic endothelial cell accumulation of fCNT was imaged using dual immunohistochemical staining of mouse lymph nodes with anti-AF488 to locate fCNT and anti-Lyve1 to mark the specialized endothelium (S6 Fig). Previously we reported that the Stabilin scavenger receptors, expressed in lymph (and also in liver and spleen) tissue, mediated endocytosis of fCNT [8]. Images of fCNT show that the signal for this nanomaterial is colocalized with these specialized endothelial cells in the lymph tissue. Here we present images of the cell type that internalizes the fCNT to confirm this finding.

### A hypothetical protein corona for water-soluble fCNT in serum

The binding of serum proteins with fCNT was examined using microscale thermophoresis to ask if the assembly of a protein corona surrounding fCNT is possible in vivo [17]. Binding isotherms for fCNT were generated (S7 Fig) and yielded K_d_ values of 14 μM and 13 μM for serum albumin and IgG binding, respectively. The mean physiologic concentration of serum albumin and IgG in these two primates was 661 μM and 187 μM, respectively (Tables 1 and 2). Based on this data, we expect fCNT to be in contact with sufficient concentrations of both these proteins to form a corona in serum. However, because renal elimination occurs rapidly (Figs 2–4), the interaction of these proteins with fCNT is deemed to be weak.

Furthermore, we measured a Log P value of -3.30 for fCNT using octanol/water partitioning. This quite negative value demonstrated high aqueous solubility with 3 logs more fCNT in the PBS phase than the organic phase. This result seemingly contradicts the assumption that all nanocarbon materials are hydrophobic since the functionalization process that we employ yields a quite water-soluble molecule. Indeed, this result correlates with the paradoxical biocompatibility of this material.

## Conclusions

The pharmacology of novel medicinal nanomaterials is perhaps one of the most important and least understood property of engineered biomaterials. Fibrillar nanocarbon is an excellent example of this predicament that fuels controversy with conflicting, limited data. While tremendous effort is expended in design and synthesis of nanomaterials, information describing performance in vivo is quite often incomplete, limited to rodents at best, and this deficiency slows progress in pharmaceutical development. A fundamental pillar of the biomedical engineering analysis toolkit is pharmacokinetic studies that define how an organism processes a molecular substance. Toxicological data investigates the macroscopic and microscopic pathology of tissues and blood chemistry and hematology to determine if the material is biocompatible or if there are adverse interactions with the host and evaluates the significance of its residence in vivo. Pharmacokinetic studies inform toxicological studies as knowledge of clearance and distribution assist in designing focused investigations.

Model organisms are used to execute pharmacokinetic and toxicological studies. Typically, rodents are initially employed, but in order to ultimately translate into man, it becomes necessary to study larger animal models. Nonhuman primates sufficiently parallel the biology and physiology of man and are ideal surrogates to investigate for pharmacokinetic and safety toxicology profiles. Here, we examined intravenous administration of fCNT in cynomolgus monkeys (S8–S10 Figs). Our first goal was to establish that the pharmacology that we have reported in mice translates to the primate and our second goal was to thoroughly investigate any potential untoward effects in primate kidney, liver, spleen and lymph tissue that we predicted would accumulate this material. Our final aim was to provide data that supports translation to man.

These data provide rigorous pharmacokinetic and toxicologic evaluations of the pharmacology of fCNT in nonhuman primates (S3 Table). The kidney and liver are the two major organs that accumulate and excrete [^86^Y]fCNT in primates and rodents. Each of these organ systems traffics [^86^Y]fCNT as described by two-compartment models. Quantitative pharmacokinetic analyses of nanomaterials used in combination with compartmental modeling methods has proven to be a powerful tool in predicting pharmacological [22] and toxicological [23] parameters of investigational therapeutic agents and toxicants in rodents. This nonhuman primate anatomically, biologically and physiologically parallels humans and is the closest model available to examine this novel fCNT molecular platform in vivo [24]. Our multidisciplinary effort has encompassed the synthesis, characterization, and pharmacological evaluation of fCNT in rodent and now nonhuman primate models. We now show that our blood clearance, specific tissue and cellular accumulation, and whole-body elimination findings in rodents translates to primates. Almost quantitative elimination of the fCNT occurs within several days and directly addresses a long-standing question regarding the fate of fibrillar nanocarbon in vivo. Further, we and others envision that polycationic ammonium-functionalized fibrillar nanocarbon can be deployed as a robust molecular platform to non-covalently bind and deliver small interfering RNA in gene therapy strategies [9,25,26]. Recently reported clinical success with RNA interference designed to lower systemic low-density lipoprotein in humans reinforces the concept that small interfering RNA can be directed to specific tissues using biomedical engineering approaches [27]. Our data provides strong evidence to support further evaluation of functionalized fibrillar nanocarbon for use in man, and underline the role robust pharmacological analysis in NHP can benefit nanomaterial science. These results provide important insight into the pharmacology of fibrillar nanocarbon, which brings the field of nanomedicine forward toward clinical use.

## Supporting information

S1 Fig Biodistribution of the [^86^Y]fCNT test article in mice. Five mice (balb/c, ♀, 2–3 months old, Taconic) each received an intravenous injection containing 0.03 mg and 74 kBq (0.002 mCi) of [^86^Y]fCNT via the retroorbital sinus. This correlative pharmacokinetic study used the same batch of [^86^Y]fCNT that was used in the primate study. The animals were euthanized 1 hour after administration with CO_2_ aspiration and tissue samples (blood, heart, kidneys, muscle, bone, lung, stomach, liver, spleen, brain and intestine) were harvested, weighed, and counted using a γ-counter (Packard Instrument Co.). Standards of the injected [^86^Y]fCNT were also counted to evaluate the %IA/g.
(PDF)

S2 Fig Time activity curve data. **Curves** were generated from Animal 2 dynamic PET/CT imaging data for [^86^Y]fCNT activity in the kidney, urine in bladder, liver, and blood.
(PDF)

S3 Fig Primates excrete intact [^86^Y]fCNT into the urine. Similar retention times are observed in the radiochromatograms of [^86^Y]fCNT activity in harvested primate urine (red trace) and the [^86^Y]fCNT formulation before injection into the monkeys (black trace).
(PDF)

S4 Fig Spleen histology of the two primates that received fCNT and an untreated control. Animal 1 was evaluated at 190 days for chronic effects and Animal 2 was evaluated at 14 days for acute effects (both received [^86^Y]fCNT) and a third animal that did not receive fCNT is an untreated control. Tissue was harvested at necropsy, fixed, sectioned, and stained with (**A-C**) H&E, (**D-F**) anti-CD31, (**G-I**) anti-Iba1, (**J-L**) TUNEL, and (**M-O**) Cleaved caspase 3. All scale bars are 100 μm.
(PDF)

S5 Fig Lymph node histology of the two primates that received fCNT and an untreated control. Animal 1 was evaluated at 190 days for chronic effects and Animal 2 was evaluated at 14 days for acute effects (both received [^86^Y]fCNT) and a third animal that did not receive fCNT is an untreated control. Tissue was harvested at necropsy, fixed, sectioned, and stained with (**A-C**) H&E, (**D-F**) anti-CD31, (**G-I**) anti-Iba1, (**J-L**) TUNEL, and (**M-O**) Cleaved caspase 3. The scale bars in panels **A-C** are 200 μm; the scale bars in panels **D-O** are 100 μm.
(PDF)

S6 Fig Lymphatic sinusoidal endothelial cell accumulation of fCNT was imaged using dual immunohistochemical staining (anti-AF488 and anti-Lyve1) in mouse lymph nodes. (**A**) Image overlay of fCNT (green) and Lyve1 (red) channels highlights the co-localized signals indicating sinusoidal endothelial accumulation of fCNT. Corresponding images of only the (**B**) red and (**C**) green channels. Scale bars are 500 μm for Panels **A**-**C**. (**D**) Higher magnification image of dual-stained lymph tissue that is shown in Panel **A** (scale bar is 10 μm).
(PDF)

S7 Fig Binding isotherms of fCNT and serum proteins. Microscale thermophoresis data was acquired to generate binding isotherms for fCNT and human albumin (red circles) and human IgG (blue circles). The Relative Fluorescence Units are plotted versus the concentrations of each protein.
(PDF)

S8 Fig Dynamic PET/CT whole body images of [^86^Y]fCNT in a cynomolgus monkey showing renal and bladder activity. (MPG)

S9 Fig Dynamic PET/CT axial images of [^86^Y]fCNT in a cynomolgus monkey showing renal activity. (MPG)

S10 Fig Dynamic PET/CT images of [^86^Y]fCNT in a cynomolgus monkey showing renal and hepatic processing activity. (MPG)

S1 Table Kidney rate constant values, standard deviations, coefficient of variance, and 95% confidence intervals for compartmental modeling analysis. Assume a constant 15% blood volume. The units of k are min.^-1^.
(PDF)

S2 Table Liver rate constant values, standard deviations, coefficient of variance, and 95% confidence intervals for compartmental modeling analysis. Assume a constant 20% blood volume. The units of k are min.^-1^.
(PDF)

S3 Table Animal research checklist for reporting experiments in vivo. (PDF)
