# Supplementary figures and images for: Carbon nanotubes exhibit fibrillar pharmacology in primates
Source: PLoS One. 2017 Aug 28;12(8):e0183902. doi: 10.1371/journal.pone.0183902 (PMC5573305; doi:10.1371/journal.pone.0183902)

S1

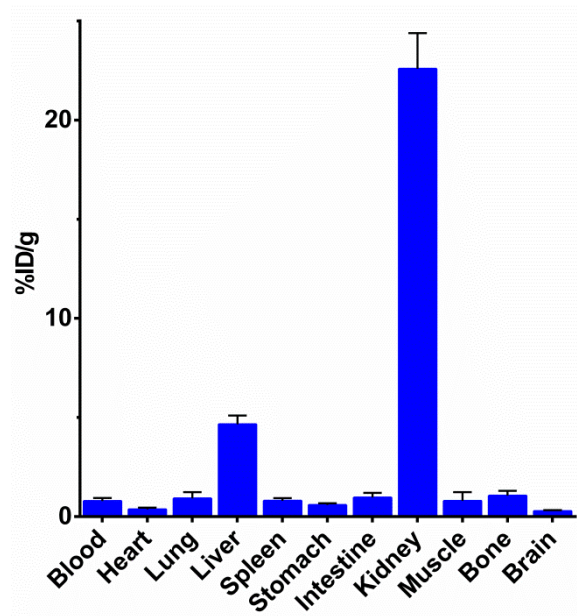

Supplement: S1 Fig — Five mice (balb/c, ♀, 2–3 months old, Taconic) each received an intravenous injection containing 0.03 mg and 74 kBq (0.002 mCi) of [86Y]fCNT via the retroorbital sinus. This correlative pharmacokinetic study used the same batch of [86Y]fCNT that was used in the primate study. The animals were euthanized 1 hour after administration with CO2 aspiration and tissue samples (blood, heart, kidneys, muscle, bone, lung, stomach, liver, spleen, brain and intestine) were harvested, weighed, and counted using a γ-counter (Packard Instrument Co.). Standards of the injected [86Y]fCNT were also counted to evaluate the %IA/g. (PDF) [file pone.0183902.s001.pdf]

S2

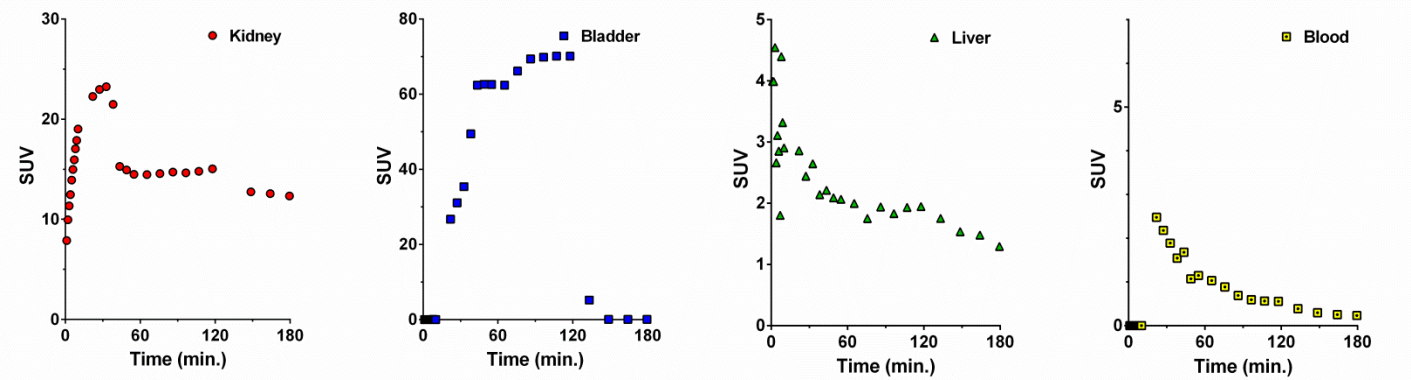

Supplement: S2 Fig — Curves were generated from Animal 2 dynamic PET/CT imaging data for [86Y]fCNT activity in the kidney, urine in bladder, liver, and blood. (PDF) [file pone.0183902.s002.pdf]

**S3**

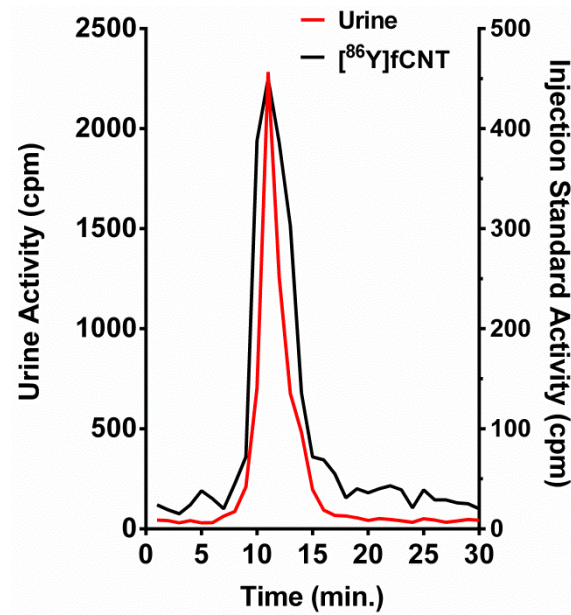

Supplement: S3 Fig — Similar retention times are observed in the radiochromatograms of [86Y]fCNT activity in harvested primate urine (red trace) and the [86Y]fCNT formulation before injection into the monkeys (black trace). (PDF) [file pone.0183902.s003.pdf]

**S4**

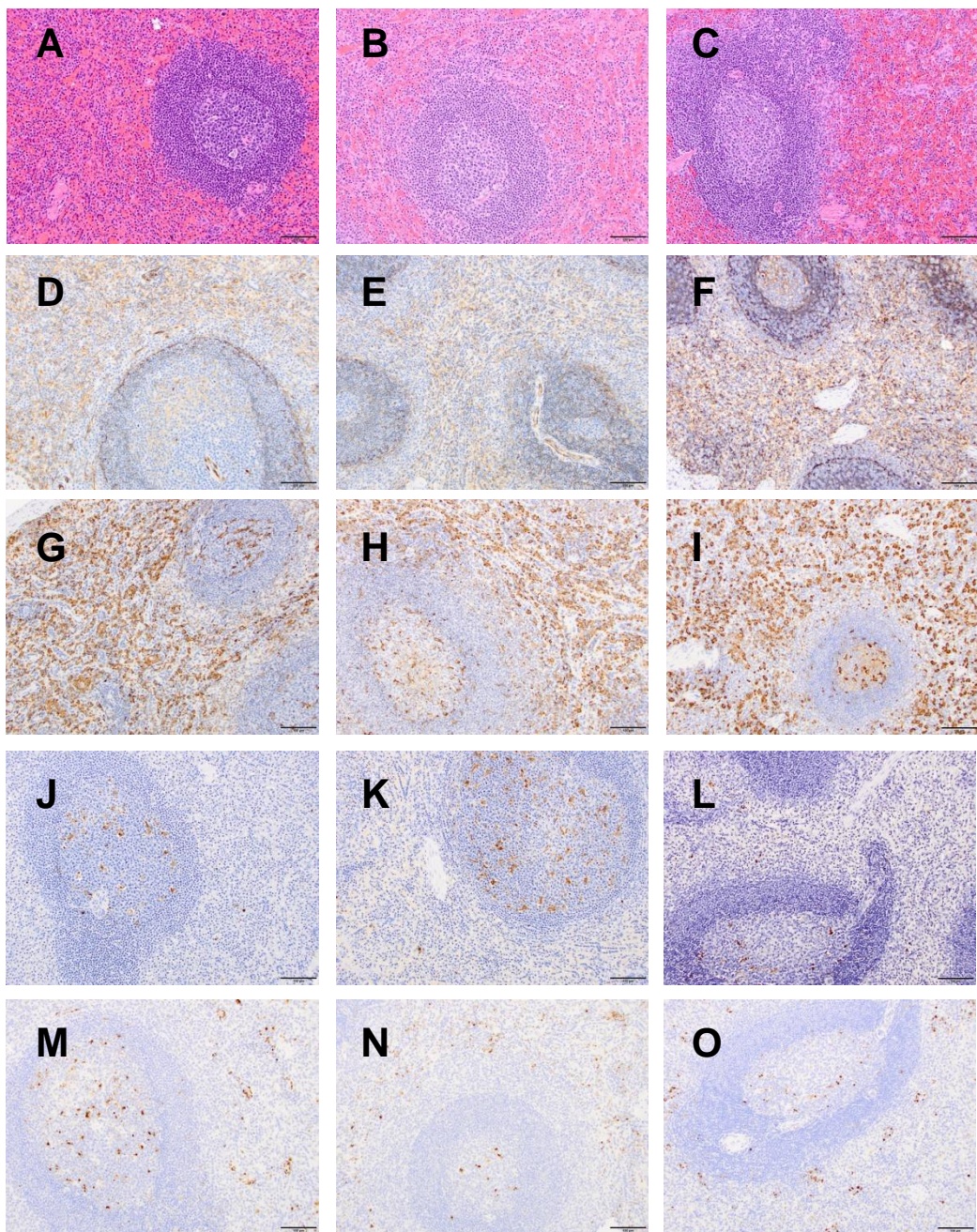

**Animal 1**

**Animal 2**

**Control**

Supplement: S4 Fig — Animal 1 was evaluated at 190 days for chronic effects and Animal 2 was evaluated at 14 days for acute effects (both received [86Y]fCNT) and a third animal that did not receive fCNT is an untreated control. Tissue was harvested at necropsy, fixed, sectioned, and stained with (A-C) H&E, (D-F) anti-CD31, (G-I) anti-Iba1, (J-L) TUNEL, and (M-O) Cleaved caspase 3. All scale bars are 100 μm. (PDF) [file pone.0183902.s004.pdf]

**S5**

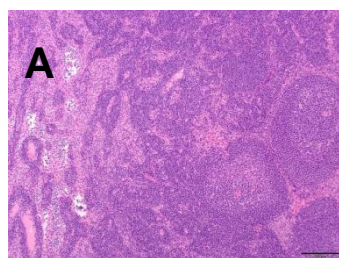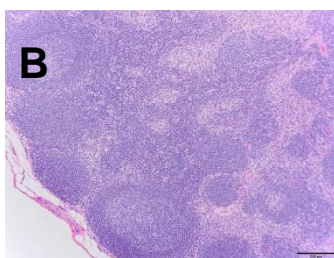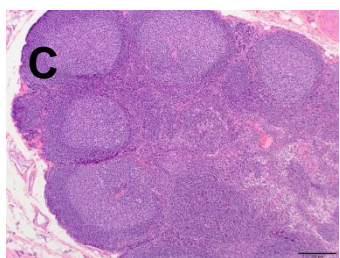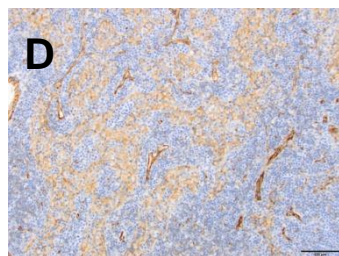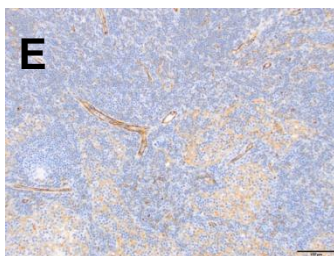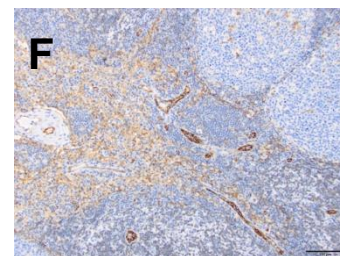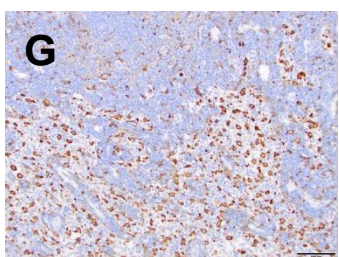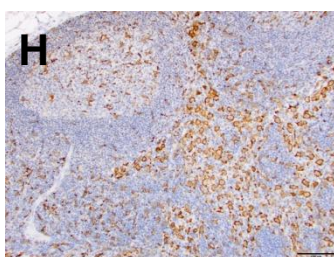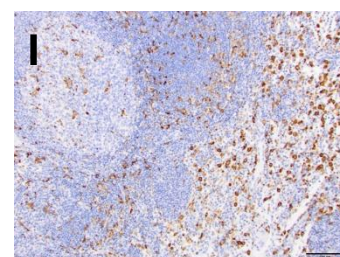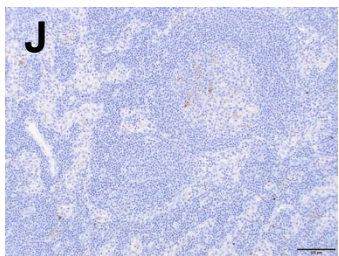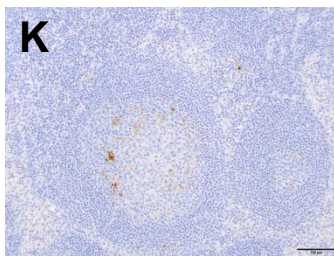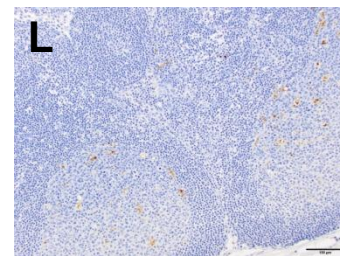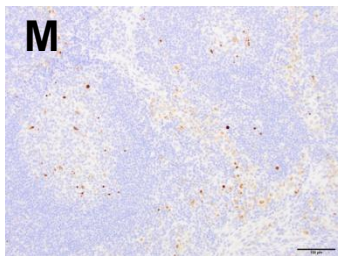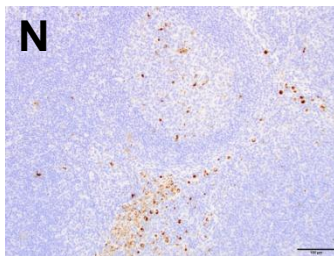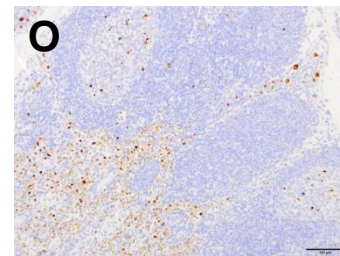

**Animal 1**

**Animal 2**

**Control**

Supplement: S5 Fig — Animal 1 was evaluated at 190 days for chronic effects and Animal 2 was evaluated at 14 days for acute effects (both received [86Y]fCNT) and a third animal that did not receive fCNT is an untreated control. Tissue was harvested at necropsy, fixed, sectioned, and stained with (A-C) H&E, (D-F) anti-CD31, (G-I) anti-Iba1, (J-L) TUNEL, and (M-O) Cleaved caspase 3. The scale bars in panels A-C are 200 μm; the scale bars in panels D-O are 100 μm. (PDF) [file pone.0183902.s005.pdf]

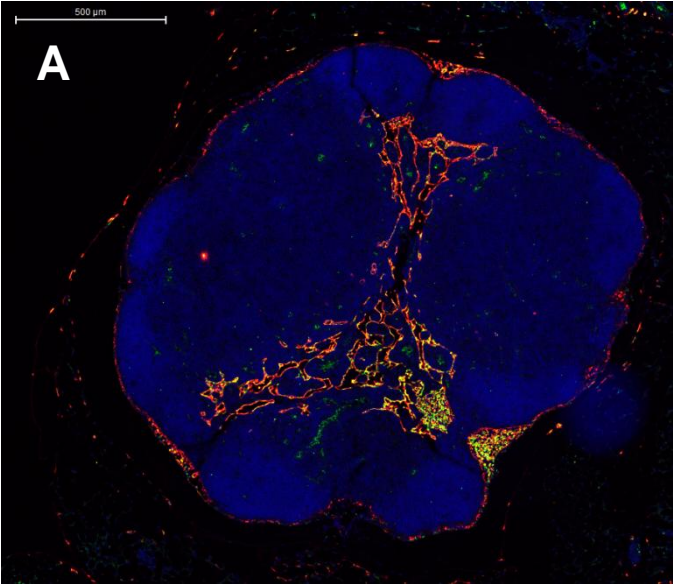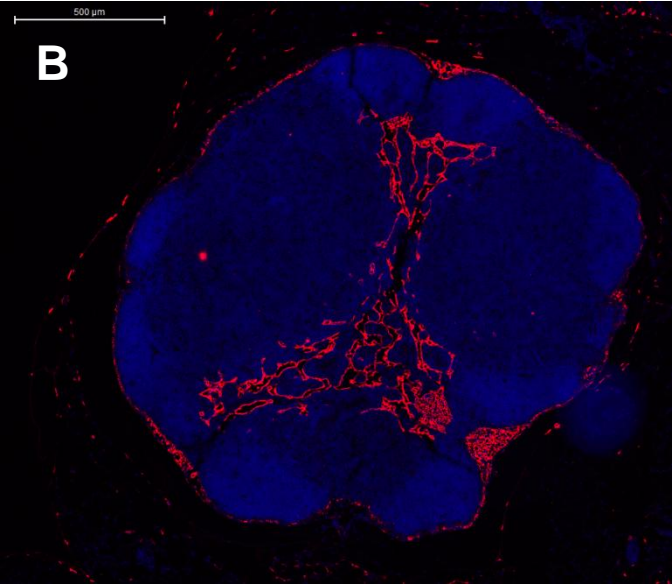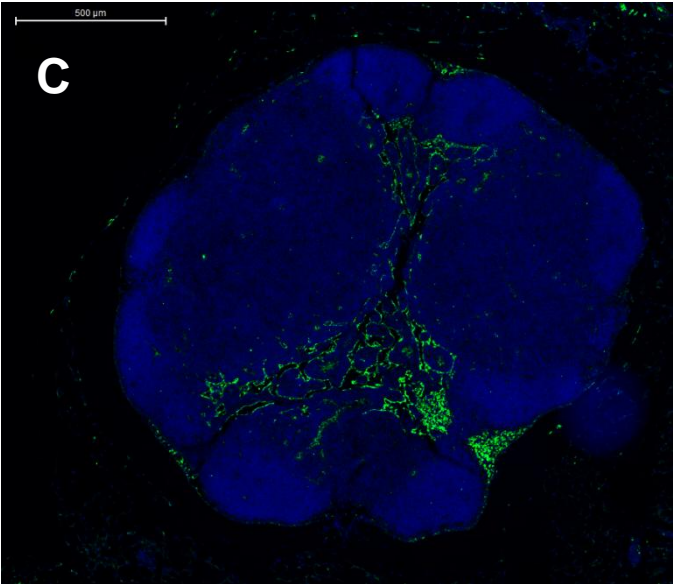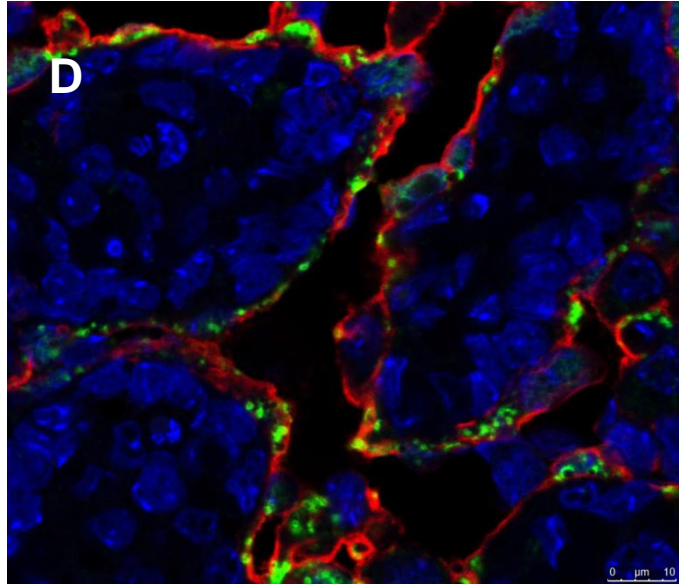

Supplement: S6 Fig — (A) Image overlay of fCNT (green) and Lyve1 (red) channels highlights the co-localized signals indicating sinusoidal endothelial accumulation of fCNT. Corresponding images of only the (B) red and (C) green channels. Scale bars are 500 μm for Panels A-C. (D) Higher magnification image of dual-stained lymph tissue that is shown in Panel A (scale bar is 10 μm). (PDF) [file pone.0183902.s006.pdf]

S7

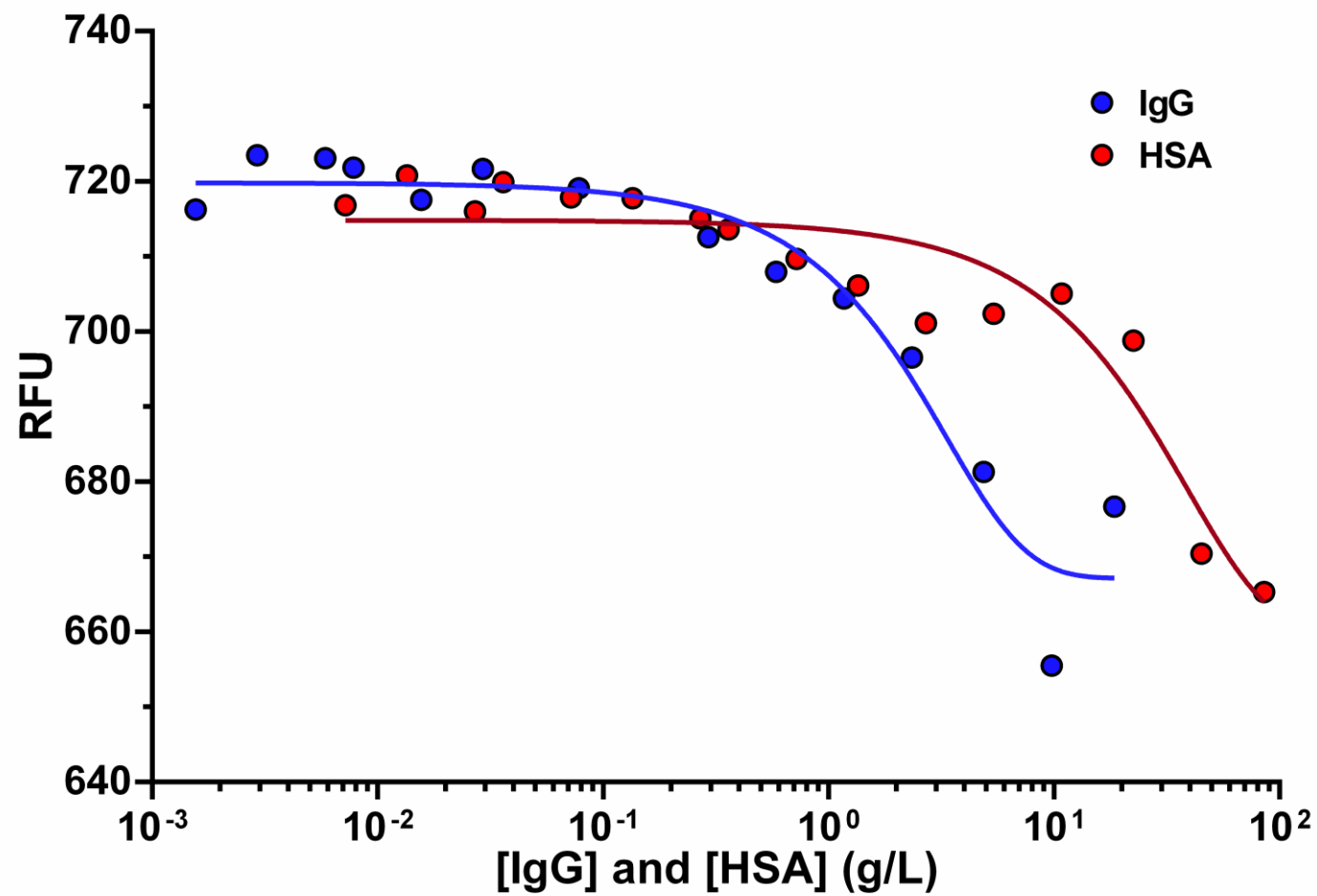

Supplement: S7 Fig — Microscale thermophoresis data was acquired to generate binding isotherms for fCNT and human albumin (red circles) and human IgG (blue circles). The Relative Fluorescence Units are plotted versus the concentrations of each protein. (PDF) [file pone.0183902.s007.pdf]
