# Supplementary material for: Carbon nanotubes exhibit fibrillar pharmacology in primates
Source: PLoS One. 2017 Aug 28;12(8):e0183902. doi: 10.1371/journal.pone.0183902 (PMC5573305; doi:10.1371/journal.pone.0183902)
Supplement: S1 Table — Assume a constant 15% blood volume. The units of k are min.-1. (PDF) [file pone.0183902.s011.pdf]

S1 Table. Renal rate constant values, standard deviations, coefficient of variance, and 95% confidence intervals for the compartmental modeling analysis. Assume a constant 15% blood volume. The units of k are min.$^{-1}$.

	**Value**	**st. dev.**	**Coeff. of var.**	**95% confidence interval**	
k_1	0.93922	6.60993e-002	7.03770e+000	0.80279	1.07564
k_2	0.15439	2.23258e-002	1.44604e+001	0.10831	0.20047
k_3	0.02875	3.78423e-003	1.31621e+001	0.02094	0.03656
k_4	0.00382	6.81979e-004	1.78658e+001	0.00241	0.00522
