# Supplementary material for: Carbon nanotubes exhibit fibrillar pharmacology in primates
Source: PLoS One. 2017 Aug 28;12(8):e0183902. doi: 10.1371/journal.pone.0183902 (PMC5573305; doi:10.1371/journal.pone.0183902)
Supplement: S2 Table — Assume a constant 20% blood volume. The units of k are min.-1. (PDF) [file pone.0183902.s012.pdf]

S2 Table. Hepatic rate constant values, standard deviations, coefficient of variance, and 95% confidence intervals for the compartmental modeling analysis. Assume a constant 20% blood volume. The units of k are $min.^{-1}$.

	**Value**	**st. dev.**	**Coeff. of var.**	**95% confidence interval**	
k_1	0.21158	3.00309e-002	1.41939e+001	0.14960	0.27356
k_2	0.41722	6.08403e-002	1.45824e+001	0.29165	0.54279
k_3	0.01003	3.82052e-004	3.80801e+000	0.00924	0.01082
k_4	0.00319	3.58493e-004	1.12348e+001	0.00245	0.00393
